# Neoadjuvant PD-1-Based Immunotherapy in Localized dMMR/MSI-H Colorectal Cancer: A Systematic Review and Arm-Based Meta-Analysis

**DOI:** 10.3390/cancers18152436

**Published:** 2026-07-29

**Authors:** Mohammed M. Alruwaili, Yehia Nabil, Yousef Alanazi, Helal G. Alanazi, Abdulrahman A. Alahmari, Emad Alqassim, Baraah Abu Alsel, Manal S. Fawzy

**Affiliations:** 1 Department of Medical Laboratory Technology, College of Applied Medical Sciences, Northern Border University, Arar 91431, Saudi Arabia; mohammed.alruwaili@nbu.edu.sa (M.M.A.); yousef.alanazi@nbu.edu.sa (Y.A.); 2 Center for Health Research, Northern Border University, Arar 91431, Saudi Arabia; 3 Faculty of Medicine, Zagazig University, Zagazig 44519, Egypt; ynabil577@gmail.com; 4 Department of Medical Laboratory, College of Applied Medical Sciences, Prince Sattam bin Abdulaziz University, Al-Kharj 11942, Saudi Arabia; h.alanazi@psau.edu.sa (H.G.A.); aa.alahmari@psau.edu.sa (A.A.A.); 5 Department of Anatomic Pathology, Pathology and Laboratory Medicine, King Faisal Specialist Hospital and Research Center, Riyadh 11211, Saudi Arabia; ealqassim@kfshrc.edu.sa; 6 Pathology Department, College of Medicine, Alfaisal University, Riyadh 11533, Saudi Arabia; 7 Medical Sciences & Preparatory Year Department, North Private College of Nursing, Arar 73244, Saudi Arabia; baraahsel@nec.edu.sa

**Keywords:** colorectal cancer, dMMR, MSI-H, neoadjuvant immunotherapy, PD-1 inhibitors, immune checkpoint blockade, pathologic complete response, major pathologic response, organ preservation, meta-analysis

## Abstract

Background/Objectives: Deficient mismatch repair (dMMR) and microsatellite instability-high (MSI-H) colorectal cancer (CRC) are highly sensitive to immune checkpoint blockade. We evaluated the efficacy, safety, and clinical maturity of neoadjuvant PD-1-based immunotherapy in localized disease. Methods: This PRISMA-compliant systematic review included prospective studies of neoadjuvant PD-1-based therapy in localized dMMR/MSI-H CRC. Proportions were pooled on the logit scale using inverse-variance random-effects models with the Paule–Mandel estimator. Pathologic complete response (pCR) was the primary outcome; major pathologic response (MPR), immune-related adverse events (irAEs), surgery, clinical complete response (cCR), organ preservation, and long-term outcomes were secondary outcomes. Results: Five prospective studies were included. Four studies, comprising 305 treated patients and 292 pathologically evaluable patients, contributed five treatment arms to the quantitative synthesis. The pooled pCR proportion was 0.65 (95% CI, 0.54–0.74), and the pooled MPR proportion was 0.89 (95% CI, 0.79–0.94). Exploratory subgroup analyses suggested higher response proportions with combination regimens, but clinical and methodological differences confounded these predominantly indirect comparisons. Any-grade irAEs occurred in 0.54 (95% CI, 0.36–0.71), whereas grade ≥3 irAEs occurred in 0.06 (95% CI, 0.04–0.10). The pooled surgery proportion was 0.96 (95% CI, 0.86–0.99), although this outcome largely reflected protocol-mandated surgery. cCR and organ preservation were promising in selected rectal-cancer cohorts. Survival and recurrence data were immature and unsuitable for quantitative pooling. Conclusions: Neoadjuvant PD-1-based immunotherapy is a highly promising investigational strategy for localized dMMR/MSI-H CRC. Nevertheless, predominantly early-phase evidence, indirect comparisons, heterogeneous endpoints, and limited follow-up preclude definitive conclusions regarding routine clinical adoption.

## Simple Summary

Some colorectal cancers have deficient DNA mismatch repair or high microsatellite instability and are particularly sensitive to PD-1-based immunotherapy. We combined evidence from five prospective studies: four studies contributing five treatment arms to the quantitative analyses and one rectal-cancer study contributing clinical complete-response and organ-preservation evidence. Pathologic complete and major pathologic responses were frequent, and severe reported immune-related adverse events were uncommon. However, the evidence remains based mainly on small, early-phase studies with heterogeneous regimens, assessment methods, surgical pathways, and limited follow-up. Clinical complete response and avoidance of surgery are especially promising in carefully selected patients with rectal cancer managed within structured watch-and-wait programs, but these outcomes should not be generalized to all localized colorectal cancers. Larger prospective comparative studies with standardized endpoints and longer oncologic surveillance are required before treatment de-escalation or organ preservation can replace established care pathways.

## 1. Introduction

Deficient mismatch repair (dMMR) and microsatellite instability-high (MSI-H) colorectal cancer (CRC) constitute a biologically distinct molecular subtype characterized by high tumor mutational burden, abundant neoantigen generation, and prominent immune-cell infiltration, features that collectively confer marked immunogenicity [1,2,3]. This subtype accounts for approximately 10–15% of nonmetastatic colorectal cancers and can be identified by immunohistochemistry for loss of mismatch repair protein expression or by molecular detection of microsatellite instability, criteria originally established by population studies informed by the Bethesda guidelines and subsequently incorporated into routine clinical and pathologic practice [4]. Curative surgical resection remains central to the management of localized disease, with adjuvant treatment selected according to pathologic stage and contemporary clinical guidance [5].

However, dMMR/MSI-H colorectal cancer appears relatively resistant to conventional cytotoxic therapy. Although neoadjuvant chemotherapy improves disease control in locally advanced colon cancer overall, pathologic regression in the dMMR subgroup of the FOxTROT trial was substantially lower than in mismatch repair-proficient tumors, highlighting the limited chemosensitivity of this molecular subtype specifically [6].

In contrast, immune checkpoint blockade has demonstrated durable clinical benefit in metastatic dMMR/MSI-H colorectal cancer, producing high response rates and significantly improving clinical outcomes compared with conventional chemotherapy [3,7]. These observations provided a compelling biological and clinical rationale for investigating immunotherapy in earlier-stage disease settings.

Recent prospective studies have demonstrated substantial activity of neoadjuvant PD-1-based immunotherapy in localized dMMR/MSI-H colorectal cancer. In colon cancer, the phase II NICHE-2 study showed that short-course nivolumab plus ipilimumab induced pathologic responses in nearly all evaluable patients, including high rates of major pathologic response (MPR) and pathologic complete response (pCR) [8]. NICHE-3 extended this signal to combined PD-1 and LAG-3 blockade with nivolumab plus relatlimab [9]. Additional prospective studies evaluating sintilimab-, prolgolimab-, and other PD-1-based regimens have reinforced the reproducibility of deep pathologic responses across localized dMMR/MSI-H colorectal cancer populations [10,11,12].

In rectal cancer, immune checkpoint blockade has also introduced the possibility of organ-preserving treatment. Cercek and colleagues reported high clinical complete response (cCR) rates with dostarlimab in carefully selected patients with locally advanced dMMR rectal cancer, allowing nonoperative management within a structured surveillance program [13,14]. This potential is clinically important because standard multimodality treatment for rectal cancer can cause long-term bowel, urinary, sexual, fertility, and stoma-related morbidity [15,16]. Nevertheless, cCR and organ preservation are distinct from histopathologically confirmed response and should not be generalized from selected rectal-cancer cohorts to all localized colorectal cancers.

Despite these encouraging findings, the evidence remains difficult to synthesize because most studies are early-phase and single-arm, and because treatment regimens, tumor sites, response-assessment frameworks, surgical pathways, and follow-up durations differ. Pathologic complete response and clinical complete response are clinically distinct endpoints: pCR requires histopathologic confirmation after resection, whereas cCR is determined through clinical, endoscopic, and radiologic assessment within a nonoperative surveillance strategy. Accordingly, an arm-based meta-analysis of proportions is appropriate for estimating outcome frequencies, but it cannot establish comparative efficacy between regimens.

Therefore, we conducted a systematic review and arm-based meta-analysis to evaluate the efficacy and safety of neoadjuvant PD-1–based immune checkpoint blockade in localized dMMR/MSI-H colorectal cancer.

## 2. Materials and Methods

### 2.1. Study Design and Registration

This systematic review and arm-based meta-analysis of proportions was conducted in accordance with the Preferred Reporting Items for Systematic Reviews and Meta-Analyses (PRISMA) 2020 statement. An updated protocol was developed a priori, and the review was registered (Registration No. 10.17605/OSF.IO/FX5ZV) [17].

### 2.2. Eligibility Criteria

We included prospective studies enrolling adults with localized, nonmetastatic colorectal adenocarcinoma with confirmed dMMR or MSI-H disease treated with neoadjuvant PD-1-based immune checkpoint blockade. Eligible designs included prospective single-arm trials, randomized clinical trials, and nonrandomized comparative studies with extractable arm-level outcome data. Monotherapy and combination immune-checkpoint regimens were eligible. Regimens combined with cytotoxic chemotherapy, radiotherapy, or anti-VEGF therapy were excluded from the primary quantitative synthesis to preserve therapeutic comparability. Case reports, reviews, editorials, and duplicate or overlapping reports without unique outcome data were excluded. Conference abstracts were excluded when they lacked sufficient extractable data or when a complete peer-reviewed report of the same cohort was available.

For contextual narrative purposes only, we also summarized selected prospective reports of clinical complete response and organ preservation that did not meet the eligibility criteria for the primary systematic synthesis because they included non-colorectal tumors or treatment regimens incorporating PD-L1 blockade, chemotherapy, anti-VEGF therapy, or chemoradiotherapy. These additional contextual reports were not counted among the included studies and were not incorporated into the risk-of-bias assessment or quantitative synthesis. The 2025 Cercek publication was handled differently: it was linked to the included 2022 publication as a sequential report of the same phase II study and provided the most mature clinical data on complete response, nonoperative management, and follow-up for that study.

### 2.3. Information Sources and Study Selection

A systematic literature search was conducted in PubMed/MEDLINE, Scopus, Web of Science, and Cochrane CENTRAL from database inception to 6 March 2026. No language restrictions were applied. Controlled vocabulary and free-text terms addressed colorectal cancer, dMMR/MSI-H status, neoadjuvant therapy, and PD-1-based immunotherapy. Database-specific strategies and search yields are provided in Table S1. Reference lists of eligible studies and relevant reviews were screened for additional reports.

Two reviewers independently screened titles, abstracts, and full-text articles for eligibility. Disagreements were resolved by consensus discussion or adjudication by a third reviewer. For potentially overlapping cohorts, the most comprehensive or most mature dataset for each outcome was retained to minimize duplication bias. Multiple publications arising from the same study were linked and treated as a single study. For each outcome, the most complete and mature report was prioritized, whereas earlier reports were retained only when they provided unique, nonoverlapping information.

### 2.4. Data Extraction

Two reviewers independently extracted study design, enrollment period, sample size, tumor site, biomarker definition, disease stage, treatment regimen, surgical strategy, follow-up duration, response definitions, safety definitions, and long-term outcomes using a piloted form. Event numerators and endpoint-specific denominators were extracted at the study-arm level. Multi-arm studies were separated when outcomes were reported independently. Treated, safety, operated, pathologically evaluable, radiographically evaluable, and follow-up populations were retained separately; reasons for denominator differences are reported in Table S2.

### 2.5. Outcomes

The primary outcome was pathologic complete response (pCR) in resected or otherwise pathologically evaluable patients, as defined in each study. Major pathologic response (MPR) was generally defined as no more than 10% residual viable tumor. Clinical complete response (cCR) was defined according to study-specific clinical, endoscopic, and radiologic criteria without mandatory histopathologic confirmation. Organ preservation, or nonoperative management, was defined as avoiding primary tumor resection within a protocol-permitted watch-and-wait pathway. Study-specific response definitions and assessment methods are summarized in Table S3.

Pathologic complete response, clinical complete response, and organ preservation were maintained as separate endpoints. pCR requires histopathologic confirmation after resection, whereas cCR and organ preservation depend on multi-modal clinical assessment, selection for nonoperative management, and structured surveillance. Therefore, cCR and organ-preservation outcomes were summarized descriptively and were not pooled with pCR.

Secondary outcomes included MPR, any-grade and grade ≥3 irAEs, surgery, R0 resection among operated patients, treatment interruption or discontinuation, steroid or other immunosuppressive treatment, treatment-related surgical delay or cancellation, postoperative complications, recurrence, progression, disease-free survival (DFS), event-free survival (EFS), recurrence-free survival, and overall survival (OS), when reported. Safety outcomes were classified as immune-related, treatment-related, treatment-emergent, or all-cause according to the original study terminology and were not treated as interchangeable. For quantitative pooling, any-grade events were eligible only when the investigators reported an irAE count or explicitly assigned the reported event set to a study-defined immune-related attribution category; counts reported solely as treatment-emergent, non-immune treatment-related, or all-cause adverse events were excluded.

### 2.6. Risk of Bias and Certainty of Evidence

Risk of bias was assessed based on the study design. The randomized trial was evaluated using the Cochrane Risk of Bias 2 tool, whereas nonrandomized and single-arm studies were appraised using the Joanna Briggs Institute Critical Appraisal Checklist for Quasi-Experimental Studies. Two reviewers independently completed each assessment and resolved disagreements by consensus. Because the review primarily synthesized uncontrolled-arm proportions rather than comparative treatment effects, certainty was assessed using a structured narrative assessment informed by the GRADE domains of risk of bias, inconsistency, indirectness, imprecision, and publication bias (Table S4).

### 2.7. Statistical Analysis

We conducted separate arm-based meta-analyses of proportions for pCR, MPR, any irAE, grade ≥3 irAE, and surgery. Proportions were logit-transformed and pooled using inverse-variance weighting. Between-study variance was estimated using the Paule–Mandel estimator, and random-effects models were prespecified as the primary analyses. When an outcome contained a zero-event or all-event arm, a continuity correction of 0.5 was applied to all contributing arms. Exact binomial confidence intervals were used for individual arms. Heterogeneity was summarized using Cochran’s Q statistic, I^2^ statistic, and τ^2^ statistic. Approximate 95% prediction intervals were calculated for heterogeneous outcomes, but they were interpreted cautiously because only five treatment arms were available. Exploratory subgroup analyses compared monotherapy with combination therapy. Sensitivity analyses comprised leave-one-out analyses, exclusion of the randomized phase 1b trial (also judged to be at high risk of bias), restriction to colon-only cohorts by excluding the mixed-site study, and common-effect models. A post hoc safety sensitivity analysis restricted the any-grade irAE synthesis to arms that used explicit irAE terminology, excluding reports with mixed treatment-related/immune-related attribution frameworks. Meta-regression was not performed because only four independent quantitative studies and five treatment arms were available, which was insufficient for stable moderator estimates. Because the review relied on aggregate arm-level data, patient-level adjustment for tumor stage, nodal status, tumor burden, and biomarker characteristics was not feasible. Accordingly, no adjusted analyses were undertaken for these potential confounders. Small-study effects were not statistically assessed because fewer than 10 studies or arms contributed to each outcome.

The objective was to estimate arm-level outcome proportions rather than comparative treatment effects; therefore, eligible prospective arms were synthesized using random-effects models.

## 3. Results

### 3.1. Study Selection

**Figure 1:**
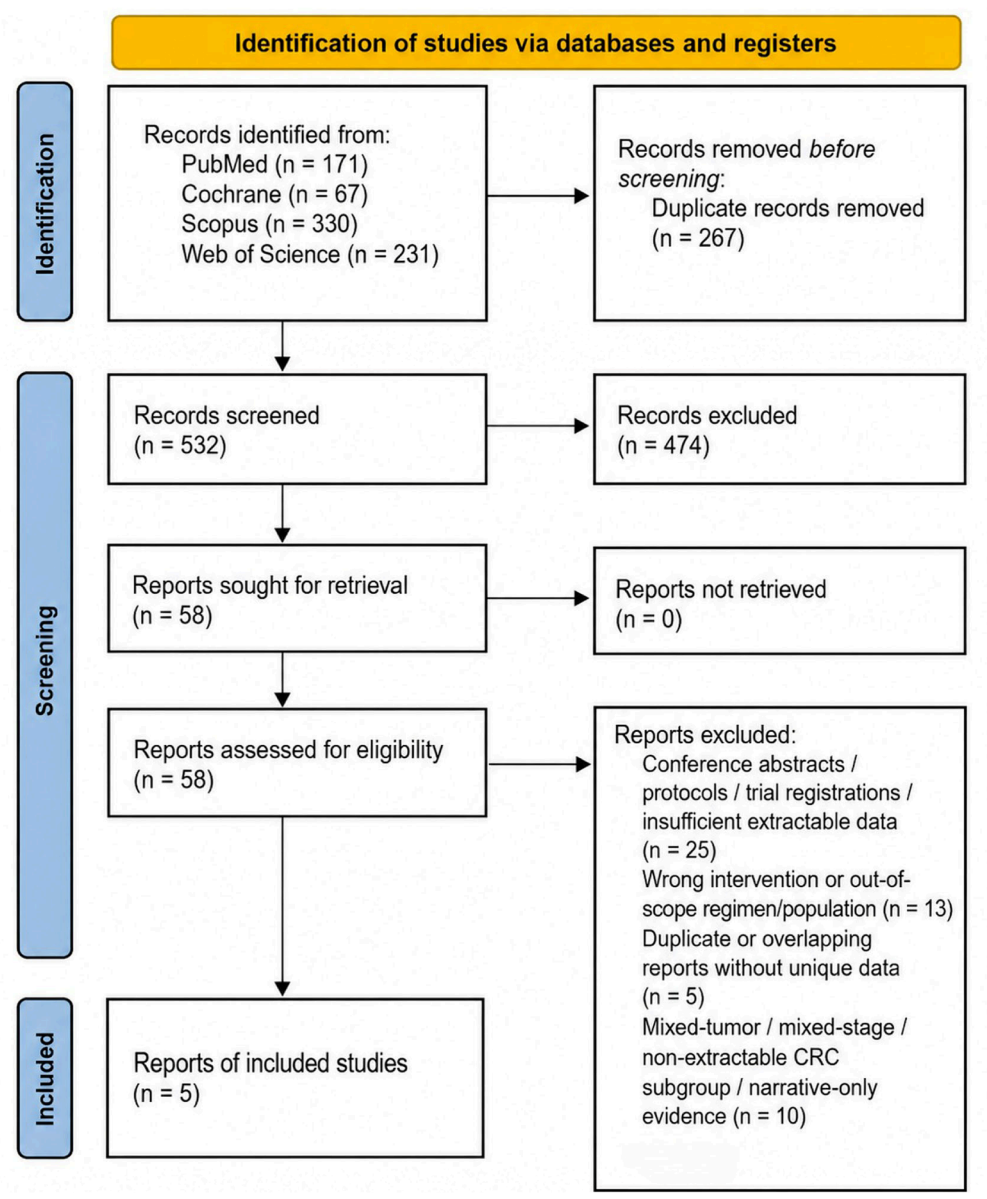
PRISMA 2020 flow diagram of study selection, showing identification of 799 records, removal of 267 duplicates, screening of 532 records, and full-text assessment of 58 reports, of which 53 were excluded, yielding 5 included studies.

The database search identified 799 records: 171 from PubMed, 67 from Cochrane CENTRAL, 330 from Scopus, and 231 from Web of Science. After removal of 267 duplicates, 532 records underwent title and abstract screening, and 58 full-text reports were assessed. Five prospective studies met the prespecified eligibility criteria. Four independent studies contributed five treatment arms to the quantitative synthesis, whereas one prospective rectal-cancer study contributed to the narrative synthesis of cCR and organ preservation (Figure 1). As detailed in the PRISMA flow diagram (Figure 1), the 53 ineligible full-text reports were excluded for the following reasons: insufficient extractable data, an ineligible intervention or population, duplicate or overlapping reporting without unique data, and absence of an extractable localized colorectal-cancer subgroup.

The Cercek 2022 [13] and Cercek 2025 [14] publications were sequential reports from the same phase II dostarlimab study and were linked and counted as one independent study. The mature 2025 report replaced the 2022 report for clinical complete response, nonoperative management, durability, recurrence-free survival, and follow-up. The earlier publication was retained only as the historical first report and for information that was not superseded by the update; it did not contain duplicate endpoint data.

### 3.2. Study and Baseline Characteristics

Five prospective studies were included overall. Four studies contributed five eligible treatment arms to the quantitative synthesis: three prospective phase II studies and one randomized, open-label, multicenter phase 1b trial with two treatment arms. The Cercek phase II dostarlimab study, as reflected in its linked 2022 and 2025 publications, contributed to the narrative synthesis of cCR, nonoperative management, and organ-preservation durability. The study and baseline characteristics are summarized in Table 1 and Table 2, respectively.

The included studies were conducted in the United States, The Netherlands, Russia, and China, and enrolled rectal-only, colon-only, or mixed colorectal cancer populations. Quantitatively synthesized regimens included nivolumab plus ipilimumab, nivolumab plus relatlimab, prolgolimab, IBI310 plus sintilimab, and sintilimab monotherapy. Dostarlimab was evaluated within a nonoperative rectal-cancer pathway. Management after neoadjuvant treatment varied substantially: most colon-cancer protocols mandated resection irrespective of clinical response, whereas selected rectal-cancer cohorts permitted watch-and-wait management after cCR.

Median follow-up ranged from 8 to 26 months in the included reports. Sample sizes ranged from 16 to 115 participants, and most cohorts included substantial proportions of participants with clinically advanced or node-positive disease. As shown in Table 2, the reported proportions of pathogenic Lynch syndrome-associated variants ranged from 19% (de Gooyer 2024) [9] to 48% (Cercek 2025) [14], reflecting differences in tumor site, completeness of hereditary testing, and population selection across studies. Molecular reporting was inconsistent across studies. Available reports variably described Lynch syndrome, sporadic dMMR, BRAF and RAS alterations, POLE/POLD1 alterations, tumor mutational burden, immune infiltration, and circulating tumor DNA. However, these biomarkers were rarely linked to endpoint-specific numerators and denominators; consequently, reliable quantitative predictor analyses were not feasible.

### 3.3. Methodological Quality Assessment

**Figure 2:**
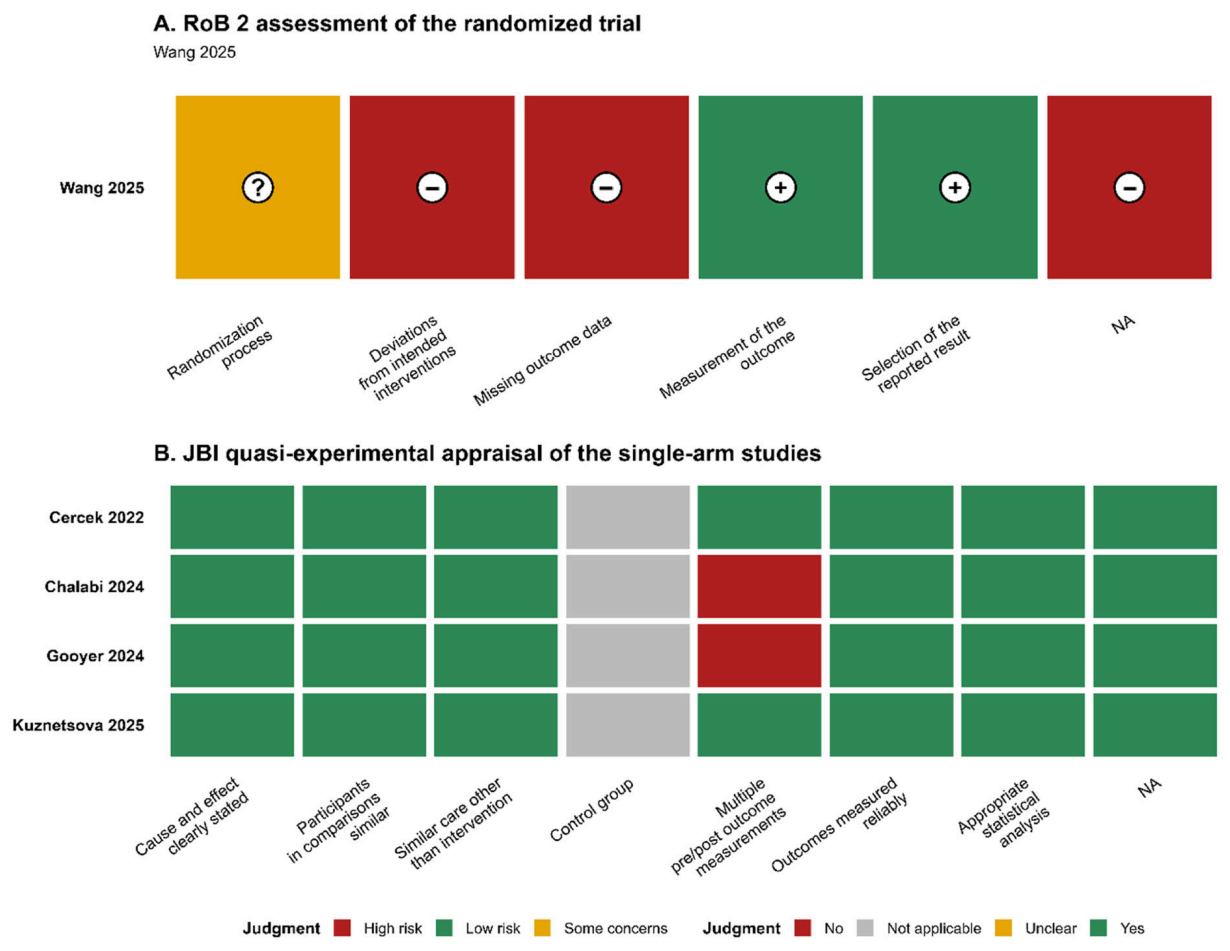
Risk-of-bias and methodological quality assessment of the included studies. (**A**) Cochrane Risk of Bias 2 (RoB 2) assessment of the randomized trial. (**B**) Joanna Briggs Institute (JBI) quasi-experimental appraisal of the single-arm studies.

The randomized phase 1b trial was judged to have some concerns regarding the randomization process and a high risk of bias owing to deviations from intended interventions and missing outcome data; the outcome measurement and selection of the reported result were judged to be at low risk (Figure 2A). Because this trial contributed two arms and the only direct regimen comparison, its influence was examined in a dedicated sensitivity analysis.

The single-arm studies demonstrated generally acceptable methodological quality on the JBI checklist, but their uncontrolled, open-label designs remain important sources of selection bias and limit causal inference (Figure 2B). Outcome measurement was generally reliable, although follow-up schedules, response frameworks, and repeated pre- and postintervention assessments were not uniform across studies. These design limitations were incorporated into the certainty assessment.

### 3.4. Pooled Efficacy Outcomes

The analytic population differed by endpoint. Five treatment arms from four independent studies contributed 292 pathologically evaluable patients to the pCR and MPR analyses, whereas surgery and pooled irAE analyses used 305 treated patients. Study-arm numerators, denominators, analytic populations, and reasons for endpoint-specific exclusions are presented in Table S2.

**Figure 3:**
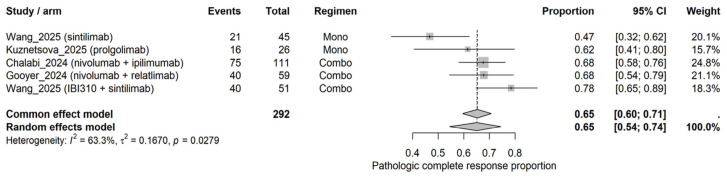
Forest plot of pathologic complete response (pCR) across five treatment arms from four independent studies (*n* = 292 pathologically evaluable patients), pooled using a random-effects model (Paule–Mandel estimator). The dot indicates “not applicable,” as the pooled common-effect estimate does not have an individual study weight. The 100.0% shown represents the total random-effects study weights.

The pooled pCR proportion was 0.65 (95% CI, 0.54–0.74), with moderate-to-substantial heterogeneity (I^2^ = 63.3%, τ^2^ = 0.1670, *p* = 0.0279) (Figure 3). The approximate 95% prediction interval was 0.43–0.82, although this interval is imprecise because only five arms were available. Individual arm estimates ranged from 0.47 with sintilimab monotherapy to 0.78 with IBI310 plus sintilimab.

**Figure 4:**
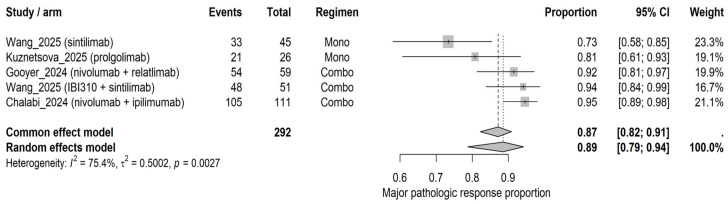
Forest plot of major pathologic response (MPR, defined as ≤10% residual viable tumor) across five treatment arms (*n* = 292 pathologically evaluable patients). The dot indicates “not applicable,” as the pooled common-effect estimate does not have an individual study weight. The 100.0% shown represents the total random-effects study weights.

The pooled MPR proportion was 0.89 (95% CI, 0.79–0.94), with substantial heterogeneity (I^2^ = 75.4%, τ^2^ = 0.5002, *p* = 0.0027) (Figure 4). The approximate 95% prediction interval was 0.62–0.97. As shown in the forest plot in Figure 4, individual arm estimates ranged from 0.73 with sintilimab monotherapy to 0.95 with nivolumab plus ipilimumab (Chalabi 2024) [8]. These results indicate frequent deep tumor regression but also substantial variation across regimens and study populations. 

### 3.5. Safety Outcomes

**Figure 5:**
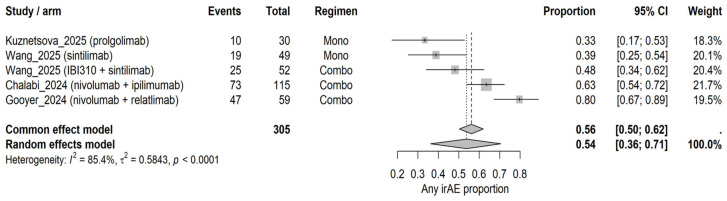
Forest plot of any-grade immune-related adverse event (irAEs) across five treatment arms (*n* = 305 treated patients); the Cercek 2025 [14] rectal-cancer cohort was excluded from this synthesis because it reported all-cause rather than immune-related adverse events. The dot indicates “not applicable”. The pooled proportion of grade ≥3 irAEs was 0.06 (95% CI, 0.04–0.10), with no detected heterogeneity (I^2^ = 0.0%, τ^2^ = 0, *p* = 0.4892) (Figure 6). Nevertheless, clinically important toxicities occurred, including hepatitis, colitis, endocrinopathies, myositis, enteritis, and myocarditis. Treatment-related surgical delays were reported in the NICHE-2, NICHE-3, and Wang combination cohorts. One patient receiving sintilimab monotherapy died from immune-mediated myocarditis, and one patient in the Wang combination arm died after postoperative anastomotic leakage and septic shock. Steroid or additional immunosuppressive treatment was required in several studies, and postoperative complication reporting was incomplete or nonuniform (Table S3A).

Pooled irAE analyses included 305 treated patients across five arms. The pooled proportion of any irAE was 0.54 (95% CI, 0.36–0.71), with considerable heterogeneity (I^2^ = 85.4%, τ^2^ = 0.5843, *p* < 0.0001) (Figure 5); the approximate 95% prediction interval was 0.18–0.86. Definitions and attribution varied across studies. We pooled only counts designated by the original investigators as irAEs or assigned to a study-defined immune-related attribution category; treatment-emergent events, non-immune treatment-related events, and all-cause adverse events were not substituted for irAEs. The Cercek report was therefore excluded because it reported all-cause adverse events rather than an irAE count. Given residual variation across attribution frameworks, the pooled result was interpreted as study-defined immune toxicity and examined in a more stringent sensitivity analysis. As shown in Figure 5, individual arm estimates for any-grade irAE ranged from 0.33 with prolgolimab monotherapy to 0.80 with nivolumab plus relatlimab, illustrating the considerable heterogeneity noted above. Study-specific safety definitions, including the rationale for excluding the Cercek all-cause adverse-event dataset [14] from this synthesis, are summarized in Table S3A.

### 3.6. Surgical Outcomes

**Figure 7:**
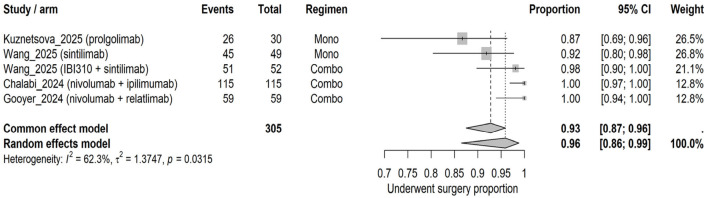
Forest plot of the proportion of patients undergoing surgery across five treatment arms (*n* = 305 treated patients); the high pooled proportion (96%) predominantly reflects protocol-mandated resection pathways in colon-cancer studies rather than a comparative measure of organ preservation. The dot indicates “not applicable”.

Surgery data were available for 305 treated patients across five arms. The pooled proportion undergoing resection was 0.96 (95% CI, 0.86–0.99), with moderate heterogeneity (I^2^ = 62.3%, τ^2^ = 1.3747, *p* = 0.0315) (Figure 7). As shown in Figure 7, individual arm estimates ranged from 0.87 with prolgolimab monotherapy to 1.00 with nivolumab plus ipilimumab (Chalabi 2024) [8] and nivolumab plus relatlimab (de Gooyer 2024) [9], the latter two reflecting protocol-mandated resection regardless of clinical response. The approximate 95% prediction interval was 0.62–1.00. R0 resection was reported in 296 of 296 operated patients.

The high surgery proportion largely reflected protocol design: most colon-cancer studies mandated resection irrespective of response. It should therefore not be interpreted as evidence of comparative efficacy, surgical necessity after immunotherapy, or the inverse of organ preservation. Organ-preservation potential must be evaluated separately in cohorts in which nonoperative management was prospectively permitted.

### 3.7. Clinical Complete Response and Organ-Preservation Outcomes

cCR and organ-preservation outcomes were analyzed separately from pCR because they depended on clinical, endoscopic, and radiologic assessment and on eligibility for structured nonoperative surveillance. The strongest evidence arose from selected patients with dMMR rectal cancer; these findings should not be generalized to all localized colorectal cancers. Of the six arms summarized in Table 3, only the Cercek 2025 [14] rectal-cancer row represents mature, included-study follow-up data; the remaining studies are presented strictly as supportive contextual evidence from tumor-agnostic cohorts or regimens incorporating PD-L1 blockade, chemotherapy, anti-VEGF therapy, or chemoradiotherapy, and were not incorporated into the quantitative synthesis.

Within the included Cercek phase II study, the updated 2025 publication superseded the 2022 report for cCR, nonoperative management, and follow-up outcomes. All 49 rectal-cancer patients who completed treatment achieved cCR and elected nonoperative management; 37 had a sustained cCR at 12 months. This updated report was not counted as an additional study and remained outside the quantitative pCR synthesis because response was assessed nonoperatively. Other reports provided supportive but clinically heterogeneous contextual evidence, including nonrectal or tumor-agnostic cohorts, PD-L1 blockade, and regimens containing chemotherapy, anti-VEGF therapy, or chemoradiotherapy. Differences in selection, cCR definitions, surveillance intensity, and follow-up precluded pooling. Descriptive evidence is presented in Table 3, while study-specific definitions are summarized in Table S3B.

Organ preservation should therefore be viewed as a promising response-adapted strategy for carefully selected rectal-cancer patients treated within multi-disciplinary watch-and-wait programs, rather than as a general conclusion for localized colorectal cancer.

### 3.8. Long-Term Oncologic Outcomes and Durability

Long-term outcomes were reported inconsistently and remained immature. In the updated dostarlimab report, the rectal-cancer cohort had a 2-year recurrence-free survival of 96% (95% CI, 90–100) at a median recurrence follow-up of 30.2 months, and no deaths occurred in either study cohort [14]. In NICHE-2, no recurrence was observed at a median follow-up of 26 months. NICHE-3 reported one recurrence among 59 patients at a median follow-up of 8 months, with all patients alive. In the prolgolimab study, two confirmed progression events occurred; 18-month DFS and OS were each 90%, and three deaths were reported, none considered treatment-related. In the randomized Wang trial, no recurrence occurred at a median follow-up of 21.4 months; however, one death occurred in each arm, and EFS and OS remained immature.

DFS, EFS, recurrence, death, and follow-up were defined and reported using different time origins and formats, and compatible hazard ratios were not available. These outcomes were therefore summarized narratively rather than pooled. Study-specific follow-up, recurrence, survival, organ-preservation durability, and salvage information are provided in Table S5.

### 3.9. Exploratory Subgroup Analysis by Regimen Type

**Figure 8:**
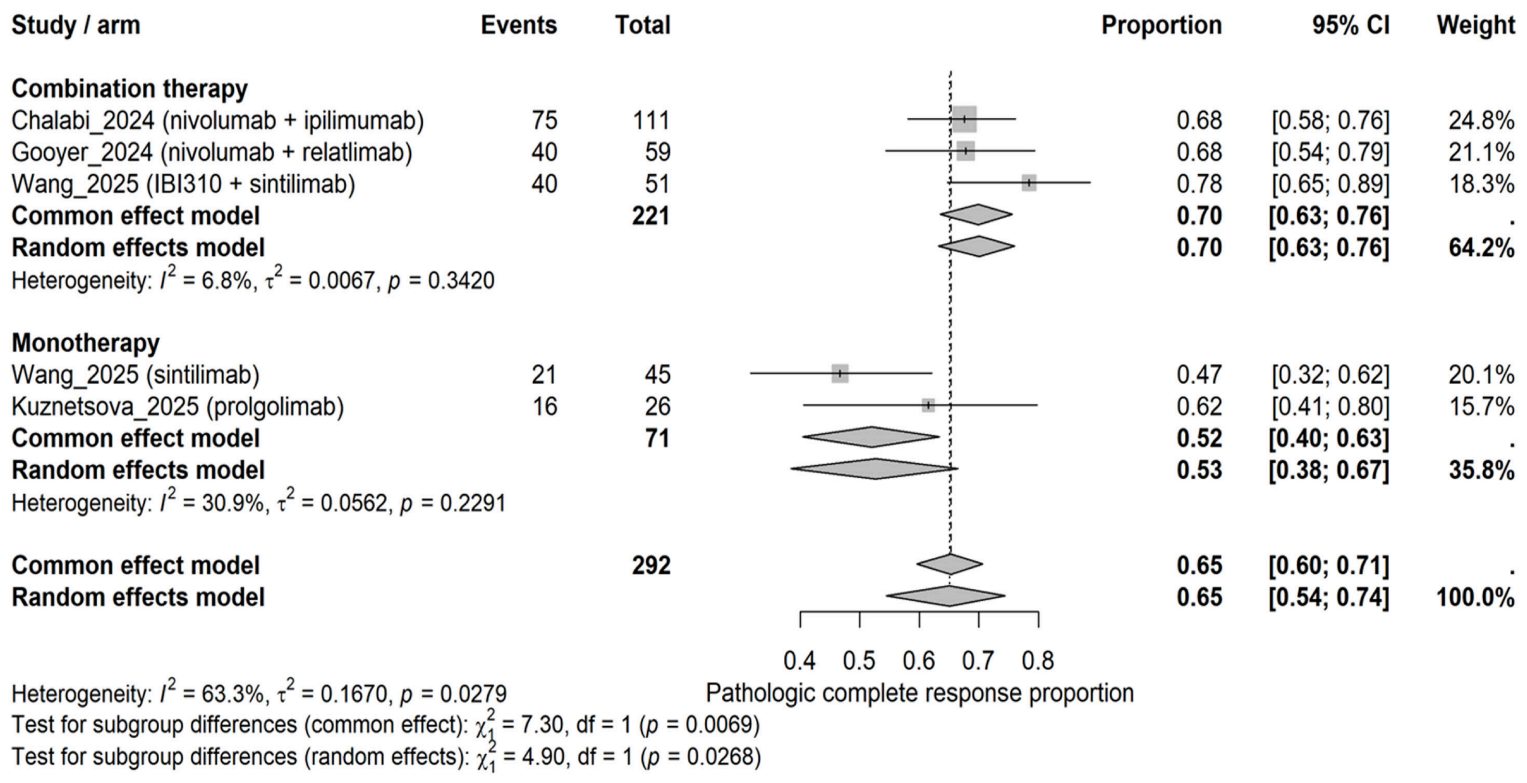
Exploratory subgroup analysis of pathologic complete response (pCR) according to regimen type (combination vs. monotherapy); comparisons are predominantly indirect between-study contrasts and should be interpreted as hypothesis-generating. The dot indicates “not applicable”.

Exploratory subgroup analyses suggested higher pooled pCR proportions with combination therapy than with monotherapy (0.70 [95% CI, 0.63–0.76] vs. 0.53 [95% CI, 0.38–0.67]; random-effects subgroup *p* = 0.0268) (Figure 8). Within Wang 2025 [10], the comparison of IBI310 plus sintilimab versus sintilimab monotherapy represented the only direct randomized comparison. All remaining regimen-type contrasts were indirect between-study comparisons and may have been confounded by differences in tumor site, study design, treatment duration, surgical strategy, and response assessment.

**Figure 9:**
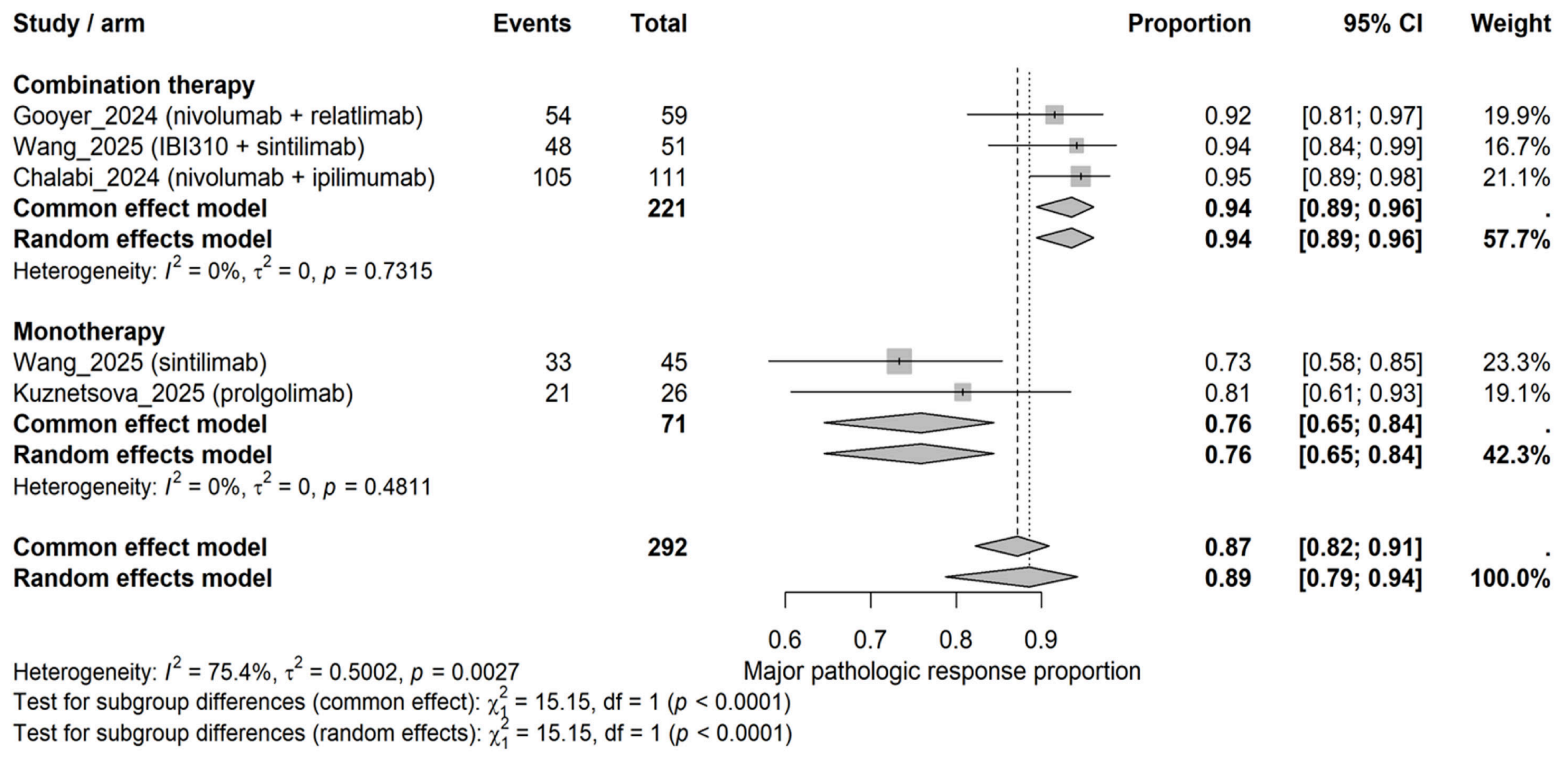
Exploratory subgroup analysis of major pathologic response (MPR) according to regimen type. The dot indicates “not applicable”.

As shown in the exploratory subgroup analysis in Figure 9, a similar pattern to that observed for pCR was seen for MPR, with pooled proportions of 0.94 (95% CI, 0.89–0.96) for combination therapy and 0.76 (95% CI, 0.65–0.84) for monotherapy (random-effects subgroup *p* < 0.0001). These estimates should be interpreted as hypothesis-generating rather than as evidence of definitive comparative superiority.

**Figure 10:**
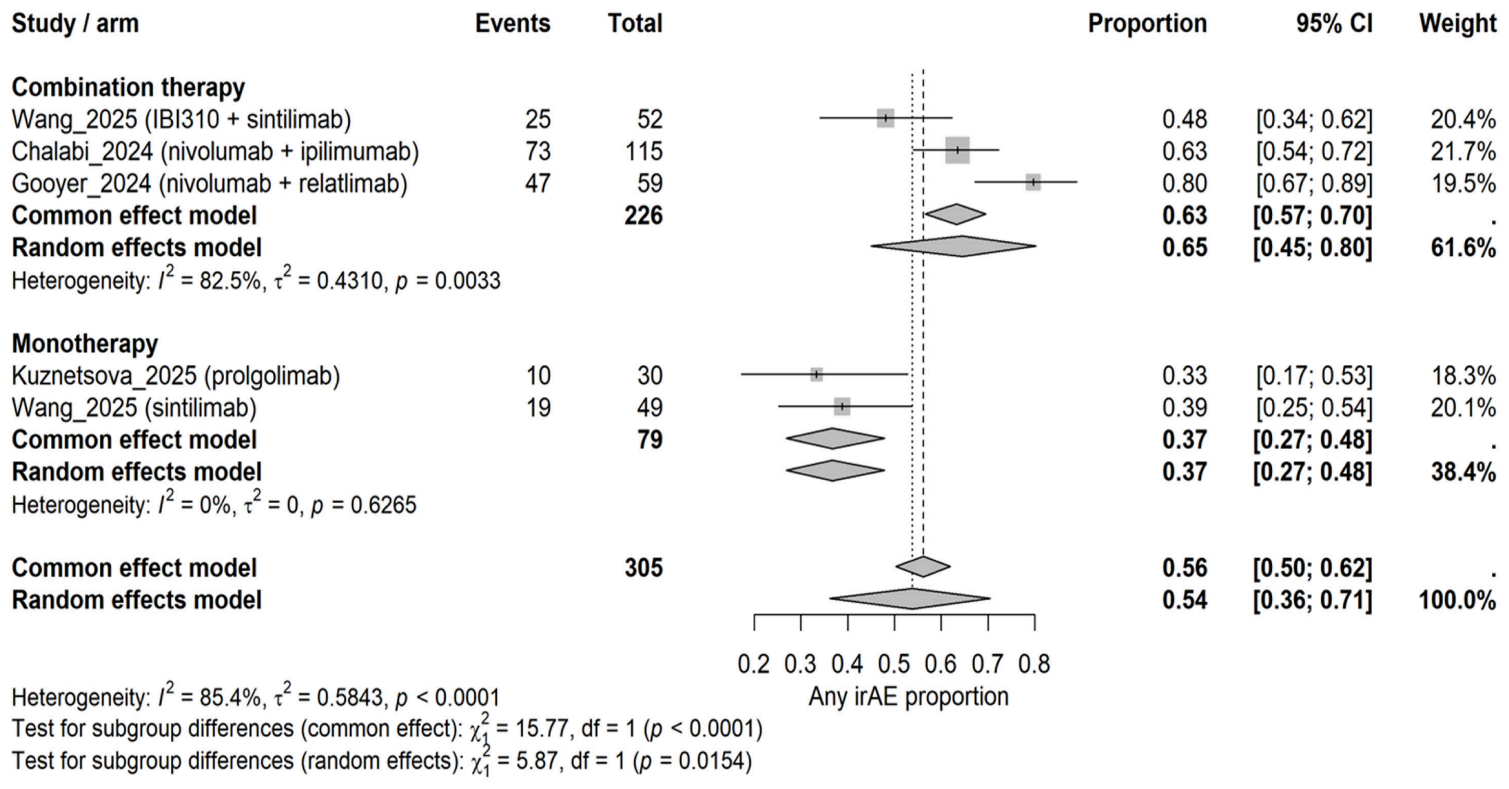
Exploratory subgroup analysis of any immune-related adverse event (irAE) according to regimen type. The dot indicates “not applicable”.

Combination regimens also appeared to have a higher pooled proportion of any irAE than monotherapy (0.65 [95% CI, 0.45–0.80] vs. 0.37 [95% CI, 0.27–0.48]; random-effects subgroup *p* = 0.0154) (Figure 10). Safety definitions and ascertainment differed across studies, limiting cross-study comparison.

**Figure 11:**
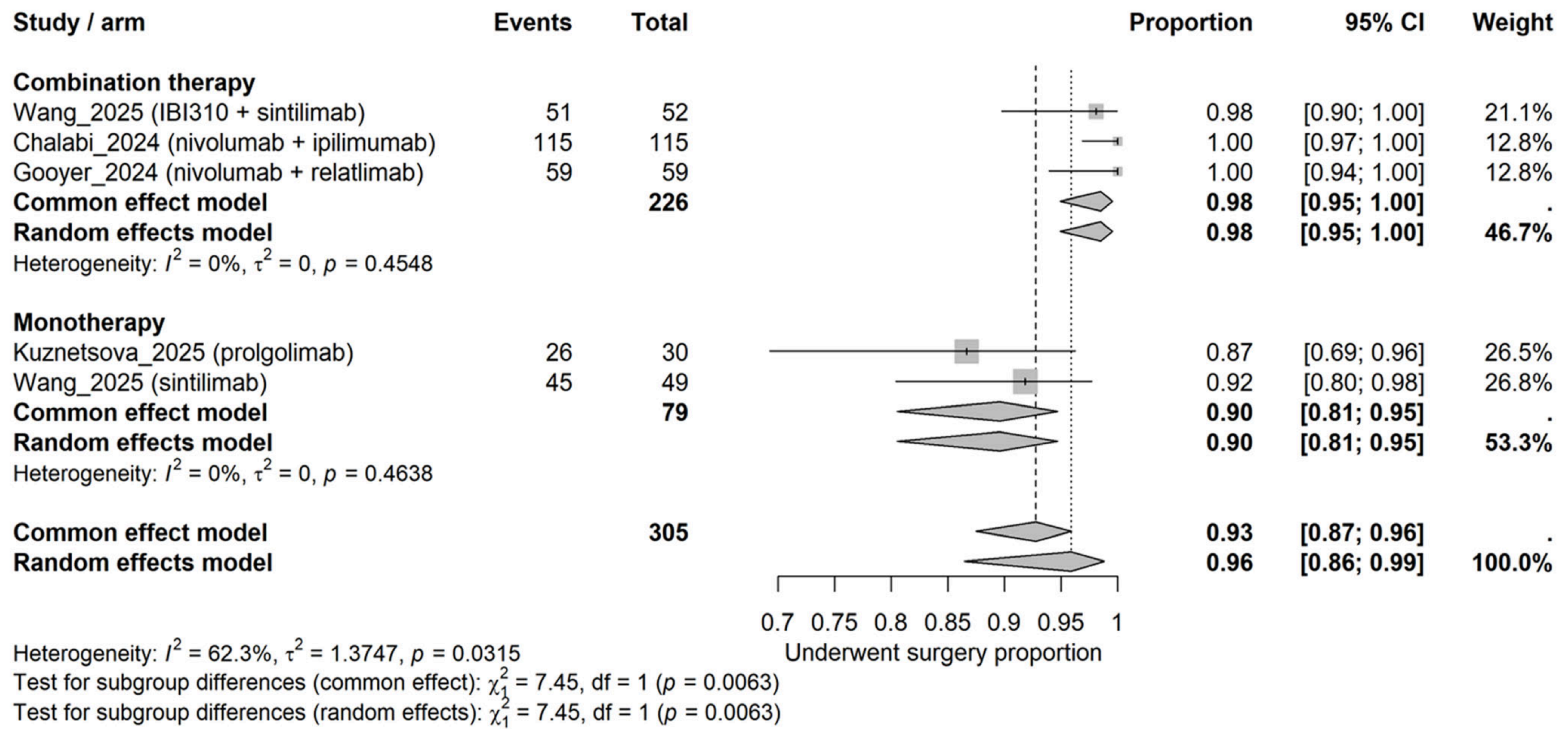
Exploratory subgroup analysis of the proportion undergoing surgery according to regimen type; the higher surgery proportion in combination-therapy arms primarily reflects protocol-mandated surgical pathways in the contributing colon-cancer studies rather than differential organ-preservation potential. The dot indicates “not applicable”.

The pooled surgery proportion was 0.98 (95% CI, 0.95–1.00) in combination arms and 0.90 (95% CI, 0.81–0.95) in monotherapy arms (random-effects subgroup *p* = 0.0063) (Figure 11). This difference primarily reflects protocol-mandated surgical pathways and should not be interpreted as a comparative measure of organ-preservation potential.

Overall, the regimen subgroup analyses identify a possible signal of higher pathologic response with combination therapy, along with greater all-grade immune toxicity. Because these analyses rely on few arms and substantial between-study differences, they are exploratory and do not replace direct randomized comparisons.

### 3.10. Sensitivity Analyses

Leave-one-out analyses showed no qualitative change in the primary conclusions: pooled pCR estimates ranged from 0.62 to 0.69 (Figure S1), MPR estimates from 0.86 to 0.91 (Figure S2), and any-irAE estimates from 0.47 to 0.58 (Figure S3). After excluding the randomized phase 1b Wang trial, which was also judged at high risk of bias, the pooled pCR proportion was 0.67 (95% CI, 0.60–0.73; I^2^ = 0%), MPR was 0.91 (95% CI, 0.81–0.96; I^2^ = 58.7%), and any irAE was 0.61 (95% CI, 0.33–0.83; I^2^ = 88.0%) (Figures S4–S6). In the colon-only analysis excluding the mixed colon/rectal prolgolimab cohort, pooled pCR was 0.66 (95% CI, 0.52–0.77; I^2^ = 72.0%), MPR was 0.90 (95% CI, 0.79–0.96; I^2^ = 80.2%), and any irAE was 0.58 (95% CI, 0.39–0.75; I^2^ = 85.6%) (Figures S7–S9). A post hoc sensitivity analysis restricted to arms that explicitly reported irAEs (Chalabi 2024 [8] and both Wang 2025 arms [10,21]; three arms, 216 patients) yielded an any-grade irAE proportion of 0.51 (95% CI, 0.37–0.65; I^2^ = 78.4%), which was consistent with the primary estimate. Common-effect models yielded directionally consistent conclusions. These analyses indicate that the response signal was robust, whereas heterogeneity in MPR and safety remained incompletely explained.

### 3.11. Certainty of Evidence

The certainty of evidence was judged very low for pCR, MPR, irAEs, surgery, cCR/organ preservation, and long-term oncologic outcomes. Downgrading reflected predominantly single-arm early-phase designs, risk of selection bias, inconsistency, indirect comparisons, small numbers of studies and events, heterogeneous endpoint definitions, and immature follow-up. The structured outcome-level assessment is presented in Table S4.

## 4. Discussion

This systematic review found that neoadjuvant PD-1-based immunotherapy was associated with frequent deep pathologic responses in localized dMMR/MSI-H colorectal cancer. The pooled pCR and MPR proportions were 65% and 89%, respectively, and the pooled proportion of grade ≥3 irAEs was 6%. These estimates are clinically compelling but should be interpreted in the context of very low-certainty evidence derived predominantly from small, early-phase, single-arm studies. The 96% pooled surgery proportion primarily reflected protocol-mandated resection and cannot be interpreted as the inverse of organ preservation or as a comparative efficacy endpoint.

The pooled findings are consistent with the major prospective studies that have defined this field. In dMMR rectal cancer, the early dostarlimab study established proof of concept for cCR-guided nonoperative management [13], and the mature report strengthened the durability signal in a larger cohort [14]. In colon cancer, NICHE-2 reported MPR in 95% and pCR in 68% after short-course nivolumab plus ipilimumab [8], while NICHE-3 reported a 97% overall pathologic response and 68% pCR with nivolumab plus relatlimab [9]. The randomized phase 1b Wang trial provided the only direct regimen comparison and favored IBI310 plus sintilimab over sintilimab monotherapy for pCR, although its phase, risk-of-bias profile, and immature survival data limit definitive inference [10]. Prolgolimab and pembrolizumab studies provide additional support for substantial activity with PD-1 monotherapy [11,18,19].

These response rates contrast with the limited pathologic regression observed with neoadjuvant chemotherapy in dMMR colon cancer in FOxTROT [6]. The contrast is biologically plausible given the immunogenicity of dMMR/MSI-H tumors. In rectal cancer, the potential clinical value extends beyond tumor regression because avoiding chemoradiotherapy and major resection may reduce long-term bowel, urinary, sexual, fertility, and stoma-related morbidity [15,16]. However, such de-escalation requires rigorous confirmation of cCR and sustained multi-disciplinary surveillance.

Organ preservation should be considered primarily in carefully selected rectal cancer cohorts managed within structured watch-and-wait programs. The mature dostarlimab platform reported cCR in all treated rectal-cancer patients and a favorable recurrence-free survival signal [14], while pembrolizumab studies also support response-adapted management in selected localized dMMR/MSI-H tumors [18,19]. Nevertheless, cCR is not equivalent to pCR, and residual or nodal disease may be missed by imaging or endoscopy. Future strategies will likely require integrating rectal MRI, endoscopy, digital examination, biopsy when appropriate, and molecular monitoring, such as circulating tumor DNA, rather than relying on a single assessment modality [19,23,24].

The apparent advantage of combination therapy in the exploratory subgroup analyses is biologically and clinically interesting but cannot be interpreted as definitive comparative efficacy. Most comparisons were indirect and confounded by differences in tumor location, baseline risk, treatment duration, checkpoint targets, surgical strategy, and outcome assessment [25,26]. Similar confounding in indirect regimen comparisons has been demonstrated in network meta-analyses of immune combination therapies in other solid tumors. The direct Wang comparison favored dual CTLA-4/PD-1 blockade for pCR [10], but the trial was a phase 1b study, had a high risk of bias in important domains, and had immature EFS and OS. Thus, combination therapy appeared to be associated with higher response proportions, but the balance between incremental efficacy, immune toxicity, cost, and complexity remains unresolved [27,28].

The expanded sensitivity analyses did not identify a single dominant explanation for heterogeneity. Excluding the Wang trial eliminated pCR heterogeneity but did not resolve heterogeneity in MPR or any irAE, whereas restriction to colon-only cohorts did not materially reduce heterogeneity. This pattern suggests that multiple correlated factors, including regimen, treatment duration, design, baseline disease burden, response assessment, and toxicity attribution, contributed simultaneously [29]. Meta-regression was not undertaken because only four independent quantitative studies were available. Tumor stage, nodal status, tumor burden, and biomarkers beyond dMMR/MSI-H could not be adjusted at the patient level. TMB, POLE/POLD1 alterations, BRAF/RAS status, immune infiltration, and ctDNA were inconsistently reported and were rarely linked to endpoint-specific numerators and denominators; consequently, no reliable quantitative predictor analysis was feasible [30]. It is important to note that only one randomized trial (Wang 2025, phase 1b, judged at high risk of bias) contributed direct comparative regimen data [21]; all remaining evidence derives from single-arm, open-label studies, so arm-based pooled proportions cannot establish comparative efficacy between regimens.

Several limitations should temper interpretation. First, the evidence base was small and dominated by open-label, single-arm, early-phase studies, with only one randomized trial. Second, arm-based proportions cannot control for between-study differences or establish causal comparative efficacy [31]. Third, aggregate data prevented patient-level adjustment for stage, nodal status, tumor burden, and biomarkers. Fourth, response definitions, safety attribution, surgical pathways, and follow-up schedules varied, and severe events were infrequent. Fifth, recurrence, DFS, EFS, OS, organ-preservation durability, local regrowth, and salvage outcomes were immature or inconsistently reported. Sixth, publication bias and small-study effects could not be assessed with fewer than 10 studies. Finally, the certainty of evidence was very low across outcomes. Larger prospective comparative studies should use standardized pathologic, clinical, safety, perioperative, survival, and watch-and-wait endpoints and should report outcomes by tumor site and clinically relevant biomarker subgroups [32,33].

## 5. Conclusions

The evidence supporting neoadjuvant PD-1-based immunotherapy in localized dMMR/MSI-H colorectal cancer remains predominantly early-phase, single-arm, and of very low certainty across all assessed outcomes; only one randomized trial, judged at high risk of bias, contributed direct comparative data on the regimen. Within this limitation, the pooled pathologic complete response (65%) and major pathologic response (89%) rates were high, and the pooled grade ≥3 immune-related adverse event rate was low (6%). However, these arm-based estimates derive from uncontrolled, heterogeneous cohorts and cannot be interpreted as comparative treatment effects. The 96% pooled surgery proportion largely reflects protocol-mandated resection pathways rather than a comparative measure of organ-preservation potential. Clinical complete response and organ preservation were promising, specifically within the mature, structured watch-and-wait rectal-cancer cohort and should not be extrapolated to localized colorectal cancer more broadly. Larger prospective, comparative studies using standardized pathologic, clinical, safety, perioperative, survival, and watch-and-wait endpoints, with longer oncologic follow-up, are needed before neoadjuvant immunotherapy can be incorporated into routine treatment pathways for localized dMMR/MSI-H colorectal cancer.

## Figures and Tables

**Figure 6:**
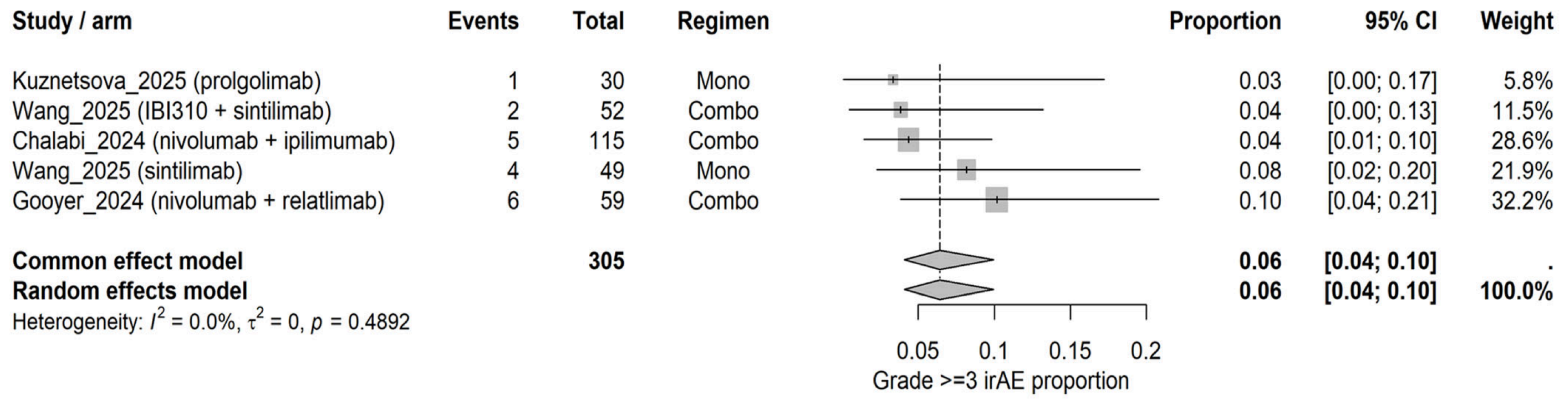
Forest plot of grade ≥3 immune-related adverse events (irAEs) across five treatment arms (*n* = 305 treated patients); no heterogeneity was detected (I^2^ = 0.0%). The dot indicates “not applicable”.

**Table 1:** Summary of the studies included.

Study, Year [Ref]	Country	Design	Population	Tumor Site	Biomarker	Neoadjuvant Regimen	Planned Management After Therapy	Follow-Up
Cercek, 2022/2025 [13,14]	USA	Prospective, phase II, single-arm; sequential reports of one study	dMMR stage I-III rectal adenocarcinoma (50 patients in the updated analysis; 49 completed treatment)	Rectum	dMMR by IHC	Dostarlimab 500 mg IV every 3 weeks for 6 months	cCR: nonoperative surveillance; residual disease: standard therapy and surgery	Median recurrence follow-up 30.2 months (range, 5.8–60.8) in the updated rectal cohort
Chalabi, 2024 [8]	The Netherlands	Phase II, multicenter, single-arm	Previously untreated dMMR colon cancer; combined NICHE and NICHE-2 cohorts (*n* = 115; efficacy *n* = 111)	Colon	dMMR by IHC	Nivolumab 3 mg/kg on days 1 and 15 plus ipilimumab 1 mg/kg on day 1	Surgery within 6 weeks of enrollment	Median 26 months
de Gooyer, 2024 [9]	The Netherlands	Phase II, multicenter, single-arm	Locally advanced resectable dMMR colon adenocarcinoma (≥T3 and/or N+) (*n* = 59)	Colon	dMMR by IHC	Nivolumab 480 mg plus relatlimab 480 mg on days 1 and 29	Surgery within 8 weeks of enrollment	Median 8 months
Kuznetsova, 2025 [11]	Russia	Prospective, open-label, nonrandomized, single-center phase II	Stage II-III dMMR/MSI colorectal adenocarcinoma (*n* = 30)	Mixed CRC	MSI by PCR; dMMR by IHC	Prolgolimab 1 mg/kg IV every 2 weeks for 12 cycles	Surgery after 6 months; treatment for one year if surgery is refused; surveillance for cCR	Median 19 months
Wang, 2025 [10]	China	Randomized, open-label, multicenter phase 1b	Previously untreated resectable MSI-H/dMMR colon cancer, stage IIB-III limited to cT4 or cN+ (*n* = 101 randomized)	Colon	MSI-H by PCR and/or dMMR by IHC	IBI310 plus sintilimab versus sintilimab alone	Curative surgery scheduled 36–56 days after the first dose	Median 21.4 months

cCR, clinical complete response; CRC, colorectal cancer; dMMR, mismatch repair deficient; IHC, immunohistochemistry; IV, intravenous; MSI, microsatellite instability; PCR, polymerase chain reaction.

**Table 2:** Baseline patient and tumor characteristics of the included study arms.

Study/Arm	*n*	Female, *n* (%)	Age, Median (Range), Years	Performance Status	Clinical T Stage	Clinical N Stage	Primary Tumor Location	Molecular/Hereditary Features
Cercek, 2025 [14]	50	28 (56)	51 (26–78)	ECOG 0: 40 (80); ECOG 1: 10 (20)	T0: 1 (2); T1-2: 10 (20); T3: 23 (46); T4: 16 (32)	Node-positive: 42 (84); node-negative: 8 (16)	Rectum only	Pathogenic Lynch syndrome-associated germline variant: 24/50 (48%); status unknown in 3/50
Chalabi, 2024 [8]	115	67 (58)	60 (20–82)	WHO 0: 100 (87); WHO 1: 15 (13)	cT2: 17 (15); cT3/4a: 24 (21); cT4a: 41 (36); cT4b: 33 (29)	cN0: 38 (33); cN+: 77 (67)	Right: 78 (68); transverse: 17 (15); left: 20 (17)	Lynch syndrome: 37 (32); unexplained dMMR: 2 (2); non-Lynch dMMR: 76 (66)
de Gooyer, 2024 [9]	59	32 (54)	65 (21–85)	WHO 0: 42 (71); WHO 1: 17 (29)	cT2: 1 (2); cT3/4a: 18 (31); cT4a: 26 (44); cT4b: 14 (24)	cN0: 22 (37); cN+: 37 (63)	Right: 48 (81); transverse: 6 (10); left: 5 (8)	Lynch syndrome: 11 (19); sporadic dMMR: 48 (81)
Kuznetsova, 2025 [11]	30	17 (57.0)	60.5 (27–82)	WHO 0: 21 (70.0); WHO 1: 9 (30.0)	cT2: 1 (3.3); cT3: 20 (66.7); cT4a: 4 (13.3); cT4b: 5 (16.7)	cN1: 27 (90.0); cN2: 3 (10.0)	Right colon: 20 (66.7); left colon: 4 (13.3); rectum: 6 (20.0)	BRAF V600E: 15 (50.0); KRAS-mutant: 10 (33.3); NRAS A59D: 1 (3.3)
Wang 2025-IBI310 + sintilimab [10]	52	23 (44.2)	56 (30–77)	ECOG 0: 22 (42.3); ECOG 1: 30 (57.7)	T2: 1 (1.9); T3: 17 (32.7); T4: 34 (65.4)	N0: 12 (23.1); N1: 26 (50.0); N2: 14 (26.9)	Left-sided: 13 (25.0); right-sided: 39 (75.0)	Non-clinically significant Lynch variant: 35 (67.3); suspected pathogenic: 3 (5.8); pathogenic: 14 (26.9)
Wang 2025-sintilimab [10]	49	22 (44.9)	56 (23–75)	ECOG 0: 24 (49.0); ECOG 1: 25 (51.0)	T3: 14 (28.6); T4: 35 (71.4)	N0: 9 (18.4); N1: 24 (49.0); N2: 16 (32.7)	Left-sided: 17 (34.7); right-sided: 32 (65.3)	Non-clinically significant Lynch variant: 34 (69.4); suspected pathogenic: 4 (8.2); pathogenic: 10 (20.4); not tested: 1 (2.0)

dMMR, mismatch repair deficient; ECOG, Eastern Cooperative Oncology Group; WHO, World Health Organization performance status.

**Table 3:** Descriptive clinical complete-response and organ-preservation evidence.

Study, Year [Ref]	Population/Tumor Site	Regimen	Organ-Preservation Pathway	cCR Assessment	cCR, *n*/N	NOM, *n*/N	Follow-Up	Interpretation
Cercek 2025 [14]	MMRd rectal cancer; mature report from the included phase II study	Dostarlimab	Patients with cCR were offered nonoperative management	No residual disease on digital and endoscopic rectal examination and absence of residual tumor on MRI	49/49	49/49	Median recurrence follow-up 30.2 months; 2-year RFS 96%	Mature included-study data; replaces the 2022 report for cCR, NOM, and follow-up; descriptive synthesis only and not pooled with pCR
Cercek 2025 [14]	Non-rectal MMRd solid tumors, including colon and other sites	Dostarlimab	Patients with cCR were offered nonoperative management	Site-specific clinical response assessment	35/54	33/54	Median recurrence follow-up 14.9 months; 2-year RFS 85%	Supportive tumor-agnostic evidence; not CRC-specific and not part of the included CRC-only dataset
Ludford 2023 [18]/LaPelusa 2025 [19]	Localized dMMR/MSI-H solid tumors; 27 CRC and 8 non-CRC	Pembrolizumab	Surgery was planned after neoadjuvant therapy, with an option for nonsurgical management and observation in selected patients.	No single CRC-specific cCR definition was consistently reported; radiographic/endoscopic response assessments were used.	NR	18/35	Median follow-up for the original nonoperative cohort: 38 weeks from last pembrolizumab; updated follow-up reported separately	Tumor-agnostic cohort; useful as supportive organ-sparing evidence, but not suitable for CRC-only pooled cCR or NOM estimates
Fang 2025 [20]	Locally advanced dMMR CRC	Envafolimab, PD-L1 blockade	Patients achieving cCR who declined surgery could enter watch-and-wait	Absence of residual disease on endoscopy and absence of residual disease on CT or MRI	2/13	2/13	Short follow-up; long-term DFS/OS not mature	Exploratory only; PD-L1 rather than PD-1 regimen; composite CR endpoint combined cCR and pCR
Wang 2025b [21]	dMMR/MSI T4NanyM0 colon cancer	Toripalimab with or without irinotecan and/or bevacizumab	Shared decision-making allowed treatment modification and the timing of surgery or watch-and-wait	Clinical complete response after treatment assessment; exact criteria were protocol-based	2/14	2/14	Median follow-up: 35.6 months	Exploratory only; regimen may include chemotherapy and anti-VEGF therapy, so it should not be included in strict PD-1-only synthesis.
Wang 2025b [21]	dMMR/MSI locally advanced rectal cancer	Toripalimab with or without irinotecan and/or bevacizumab	Rectal cancer patients could refuse upfront surgery/radiotherapy and choose watch-and-wait after cCR	Clinical complete response after 6 months of treatment	2/8	2/8	Median follow-up: 35.6 months	Exploratory rectal cancer evidence; one cCR patient later developed regional nodal progression, so interpretation should be cautious
Tsukada 2024 [22]	MSI-H locally advanced rectal cancer	Chemoradiotherapy followed by nivolumab	Protocol treatment was CRT followed by nivolumab and surgery	cCR after CRT plus nivolumab	1/5	0/5	Median follow-up: 56.4 months	Exploratory only; very small MSI-H subgroup and treatment included chemoradiotherapy before nivolumab.

cCR, clinical complete response; CRC, colorectal cancer; CRT, chemoradiotherapy; CT, computed tomography; dMMR, deficient mismatch repair; DFS, disease-free survival; MMRd, mismatch repair deficient; MRI, magnetic resonance imaging; MSI-H, microsatellite instability-high; NOM, nonoperative management; NR, not reported; OS, overall survival; pCR, pathologic complete response. The rectal-cancer row from Cercek 2025 [14] is the mature follow-up of the included Cercek phase II study [13] and replaces the 2022 report for cCR, NOM, and follow-up; it is not an additional study. The remaining rows contain only contextual narrative evidence and were not incorporated into the quantitative meta-analysis.

## Data Availability

All data supporting the findings of this review are available in the article, the cited primary reports, and the Supplementary Materials. Additional clarification may be requested from the corresponding author.

## Supplementary Materials

The following supporting information can be downloaded at https://www.mdpi.com/article/10.3390/cancers18152436/s1, Table S1, Search strategy; Table S2, Endpoint-specific numerators, denominators, and analytic populations; Table S3 Safety definitions, perioperative outcomes, and response-assessment definitions across included studies; Table S4, Structured narrative certainty assessment (GRADE-informed); Table S5, Follow-up and long-term oncologic outcomes; Figure S1, Leave-one-out sensitivity analysis for pathologic complete response (pCR); Figure S2, Leave-one-out sensitivity analysis for major pathologic response (MPR); Figure S3, Leave-one-out sensitivity analysis for any immune-related adverse event (irAE); Figure S4, Sensitivity analysis excluding the randomized phase 1b Wang 2025 trial [10], which was also the study judged at high risk of bias, for pathologic complete response (pCR); Figure S5, Sensitivity analysis excluding the randomized phase 1b Wang 2025 trial [10], which was also the study judged at high risk of bias, for major pathologic response (MPR); Figure S6, Sensitivity analysis excluding the randomized phase 1b Wang 2025 trial [10], which was also the study judged at high risk of bias, for any immune-related adverse event (irAE); Figure S7, Colon-only sensitivity analysis excluding the mixed colon/rectal Kuznetsova 2025 cohort [11] for pathologic complete response (pCR); Figure S8, Colon-only sensitivity analysis excluding the mixed colon/rectal Kuznetsova 2025 cohort [11] for major pathologic response (MPR); Figure S9, Colon-only sensitivity analysis excluding the mixed colon/rectal Kuznetsova 2025 cohort [11] for any immune-related adverse event (irAE).

## Abbreviations

The following abbreviations are used in this manuscript:

CRC	Colorectal cancer
dMMR	Deficient mismatch repair
MSI-H	Microsatellite instability-high
PD-1	Programmed cell death protein 1
CTLA-4	Cytotoxic T-lymphocyte-associated protein 4
LAG-3	Lymphocyte-activation gene 3
ICI	Immune checkpoint inhibitor
irAE	Immune-related adverse event
pCR	Pathologic complete response
MPR	Major pathologic response
cCR	Clinical complete response
OS	Overall survival
EFS	Event-free survival
TNT	Total neoadjuvant therapy
ctDNA	Circulating tumor DNA
HR	Hazard ratio
CI	Confidence interval
PRISMA	Preferred Reporting Items for Systematic Reviews and Meta-Analyses
NOM	Nonoperative management
RFS	Recurrence-free survival
JBI	Joanna Briggs Institute
RoB 2	Cochrane Risk of Bias 2
