# Supplementary material for: Neoadjuvant PD-1-Based Immunotherapy in Localized dMMR/MSI-H Colorectal Cancer: A Systematic Review and Arm-Based Meta-Analysis
Source: Cancers (Basel). 2026 Jul 29;18(15):2436. doi: 10.3390/cancers18152436 (PMC13464875; doi:10.3390/cancers18152436)
Supplement: Supplementary file 1 [file cancers-18-02436-s001.zip › cancers-4430957-supplementary.pdf]

Neoadjuvant PD-1–Based Immunotherapy in Localized dMMR/MSI-H Colorectal Cancer: A Systematic Review and Arm-Based Meta-Analysis

Table S1. Search Strategy.

Database	SS	No. of results
PubMed	("colorectal neoplasms"[MeSH] OR colorectal cancer*[tiab] OR colon cancer*[tiab] OR rectal cancer*[tiab]) AND ("Microsatellite Instability"[MeSH] OR microsatellite instab*[tiab] OR MSI-H[tiab] OR mismatch repair deficien*[tiab] OR dMMR[tiab]) AND (neoadjuvant[tiab] OR preoperative[tiab]) AND (PD-1[tiab] OR PD1[tiab] OR pembrolizumab[tiab] OR nivolumab[tiab] OR dostarlimab[tiab] OR sintilimab[tiab] OR prolgolimab[tiab] OR ipilimumab[tiab] OR relatlimab[tiab])	171
Cochrane	("colorectal neoplasms" OR "colorectal cancer*" OR "colorectal carcinoma*" OR "colon cancer*" OR "rectal cancer*" OR "colon neoplasm*" OR "rectal neoplasm*" OR CRC)<br>AND<br>("microsatellite instability" OR "microsatellite instab*" OR "MSI" OR "MSI-H" OR "mismatch repair deficien*" OR "deficient mismatch repair" OR "MMR deficient" OR dMMR)<br>AND<br>(neoadjuvant OR preoperative OR pre-operative OR "pre-operative therapy" OR "neoadjuvant therapy")<br>AND<br>("PD-1" OR PD1 OR "PD-1 inhibitor*" OR pembrolizumab OR nivolumab OR dostarlimab OR sintilimab OR prolgolimab OR ipilimumab OR relatlimab OR immunotherapy OR "immune checkpoint inhibitor*" OR "checkpoint blockade")	67
Web of science	TS=("colorectal neoplasms" OR "colorectal cancer*" OR "colon cancer*" OR "rectal cancer*")<br>AND<br>TS=("microsatellite instability" OR "microsatellite instab*" OR "MSI-H" OR "mismatch repair deficien*" OR "dMMR")<br>AND<br>TS=(neoadjuvant OR preoperative)	231

	AND<br>TS=("PD-1" OR PD1 OR pembrolizumab OR nivolumab OR dostarlimab OR sintilimab OR prolgolimab OR ipilimumab OR relatlimab)	
Scopus	(TITLE-ABS-KEY("colorectal neoplasms" OR "colorectal cancer*" OR "colon cancer*" OR "rectal cancer*"))<br>AND<br>(TITLE-ABS-KEY("microsatellite instability" OR "microsatellite instab*" OR "MSI-H" OR "mismatch repair deficien*" OR "dMMR"))<br>AND<br>(TITLE-ABS-KEY(neoadjuvant OR preoperative))<br>AND<br>(TITLE-ABS-KEY("PD-1" OR PD1 OR pembrolizumab OR nivolumab OR dostarlimab OR sintilimab OR prolgolimab OR ipilimumab OR relatlimab))	330
Total		799

Table S2 Endpoint-specific numerators, denominators, and analytic populations.

(A) Outcomes included in the main quantitative synthesis.

Study/arm	Endpoint	Events/N	Analytic population	Role	Reason for denominator
Chalabi_2024 — nivolumab_ipilimumab	Pathologic complete response	75/111	Efficacy/pathology population	Yes	Central pathologic assessment; 4 treated patients excluded from efficacy population
Chalabi_2024 — nivolumab_ipilimumab	Major pathologic response	105/111	Efficacy/pathology population	Yes	≤10% residual viable tumor
Chalabi_2024 — nivolumab_ipilimumab	Underwent surgery	115/115	Treated population	Yes	Protocol-mandated surgery
Chalabi_2024 — nivolumab_ipilimumab	Any irAE	73/115	Safety population	Yes	All treated patients
Chalabi_2024 — nivolumab_ipilimumab	Grade ≥3 irAE	5/115	Safety population	Yes	Grade 3–4 irAEs
Gooyer_2024 — nivolumab_relatlimab	Pathologic complete response	40/59	Efficacy/pathology population	Yes	All 59 evaluable
Gooyer_2024 — nivolumab_relatlimab	Major pathologic response	54/59	Efficacy/pathology population	Yes	≤10% residual viable tumor
Gooyer_2024 — nivolumab_relatlimab	Underwent surgery	59/59	Treated population	Yes	Protocol-mandated surgery
Gooyer_2024 — nivolumab_relatlimab	Any irAE	47/59	Safety population	Yes	AEs at least possibly related to nivolumab and/or relatlimab
Gooyer_2024 — nivolumab_relatlimab	Grade ≥3 irAE	6/59	Safety population	Yes	Grade 3–4 irAEs
Kuznetsova_2025 — prolgolimab	Pathologic complete response	16/26	Operated patients	Yes	Pathologic analysis restricted to operated patients
Kuznetsova_2025 — prolgolimab	Major pathologic response	21/26	Operated patients	Yes	Mandard TRG 1–2; ≤10% residual viable tumor

Study/arm	Endpoint	Events/N	Analytic population	Role	Reason for denominator
Kuznetsova_2025 — prolgolimab	Underwent surgery	26/30	Treated population	Yes	Four patients did not undergo surgery
Kuznetsova_2025 — prolgolimab	Any irAE	10/30	Safety population	Yes	All treated patients; CTCAE v5.0
Kuznetsova_2025 — prolgolimab	Grade ≥3 irAE	1/30	Safety population	Yes	Grade 4 autoimmune hepatitis
Wang_2025 — IBI310_sintilimab	Pathologic complete response	40/51	Modified ITT pathology population	Yes	One patient excluded from mITT pathological analysis
Wang_2025 — IBI310_sintilimab	Major pathologic response	48/51	Modified ITT pathology population	Yes	RVT ≤10%
Wang_2025 — IBI310_sintilimab	Underwent surgery	51/52	Treated population	Yes	One patient did not undergo surgery
Wang_2025 — IBI310_sintilimab	Any irAE	25/52	Safety population	Yes	Table 2 safety population
Wang_2025 — IBI310_sintilimab	Grade ≥3 irAE	2/52	Safety population	Yes	Results and Table 2 report 2/52; discussion text states 3 and is flagged
Wang_2025 — sintilimab	Pathologic complete response	21/45	Modified ITT pathology population	Yes	Four treated patients excluded from mITT pathological analysis
Wang_2025 — sintilimab	Major pathologic response	33/45	Modified ITT pathology population	Yes	RVT ≤10%
Wang_2025 — sintilimab	Underwent surgery	45/49	Treated population	Yes	Four patients did not undergo surgery
Wang_2025 — sintilimab	Any irAE	19/49	Safety population	Yes	Table 2 safety population

Study/arm	Endpoint	Events/N	Analytic population	Role	Reason for denominator
Wang_2025 — sintilimab	Grade ≥3 irAE	4/49	Safety population	Yes	Includes fatal immune-mediated myocarditis

(B) Descriptive and secondary endpoints.

Study/arm	Endpoint	Events/ N	Analytic population	Role	Reason for denominator
Cercek_2025 — dostarlimab	Clinical complete response	49/49	Treatment-completers / response-evaluable	No	All 49 rectal-cancer patients who completed treatment achieved cCR; mature report replaces the 2022 cCR data
Cercek_2025 — dostarlimab	Nonoperative management	49/49	Treatment-completers / response-evaluable	No	All 49 rectal-cancer treatment completers elected nonoperative management
Cercek_2025 — dostarlimab	Sustained cCR at 12 months	37/49	Treatment-completers	No	Thirty-seven patients had sustained cCR at 12 months; follow-up was still accruing for some patients
Cercek_2025 — dostarlimab	Two-year recurrence-free survival	96% (95% CI, 90-100)	Rectal-cancer analysis cohort (n=50)	No	Median recurrence follow-up 30.2 months; event counts were not separately tabulated as a simple recurrence proportion
Cercek_2025 — dostarlimab	Death	0/50	Rectal-cancer analysis cohort	No	No deaths occurred in either cohort of the updated report
Chalabi_2024 — nivolumab_ipilimumab	Any pathologic response	109/111	Efficacy/pathology population	Secondary	≤50% residual viable tumor
Chalabi_2024 — nivolumab_ipilimumab	R0 resection	115/115	Operated patients	Secondary	100% among operated patients

Study/arm	Endpoint	Events/ N	Analytic population	Role	Reason for denominator
Chalabi_2024 — nivolumab_ipilimumab	Recurrence	0/115	Follow-up population	Narrative	No recurrence at median follow-up 26 months
Gooyer_2024 — nivolumab_relatlimab	Any pathologic response	57/59	Efficacy/pathology population	Secondary	≤50% residual viable tumor
Gooyer_2024 — nivolumab_relatlimab	R0 resection	59/59	Operated patients	Secondary	100% among operated patients
Gooyer_2024 — nivolumab_relatlimab	Recurrence	1/59	Follow-up population	Narrative	58/59 disease-free at median follow-up 8 months
Gooyer_2024 — nivolumab_relatlimab	Death	0/59	Follow-up population	Narrative	All alive at report cutoff
Kuznetsova_2025 — prolgolimab	Clinical complete response	6/29	Radiographically evaluable	No	Radiographic/endoscopic cCR; five subsequently underwent surgery
Kuznetsova_2025 — prolgolimab	Objective response rate	26/29	Radiographically evaluable	No	Radiographic/endoscopic assessment
Kuznetsova_2025 — prolgolimab	Primary composite endpoint: pCR or cCR	17/30	Intention-to-treat	No	16 pCR plus one nonoperated cCR; not interchangeable with pCR
Kuznetsova_2025 — prolgolimab	R0 resection	26/26	Operated patients	Secondary	All operated cases achieved R0
Kuznetsova_2025 — prolgolimab	Confirmed progression	2/30	Follow-up population	Narrative	Do not pool directly with simple recurrence without harmonization
Kuznetsova_2025 — prolgolimab	Death	3/30	Follow-up population	Narrative	Full text reports three deaths: unrelated cardiac arrest, disease-progression death, and one unknown-cause postoperative death

Study/arm	Endpoint	Events/ N	Analytic population	Role	Reason for denominator
Wang_2025 — IBI310_sintilimab	R0 resection	51/51	Operated patients	Secondary	All operated patients achieved R0
Wang_2025 — IBI310_sintilimab	Recurrence	0/52	Follow-up population	Narrative	No recurrence at median follow-up 21.4 months
Wang_2025 — IBI310_sintilimab	Death	1/52	Follow-up population	Narrative	Postoperative anastomotic leak/septic shock leading to multiple-organ dysfunction
Wang_2025 — sintilimab	R0 resection	45/45	Operated patients	Secondary	All operated patients achieved R0
Wang_2025 — sintilimab	Recurrence	0/49	Follow-up population	Narrative	No recurrence at median follow-up 21.4 months
Wang_2025 — sintilimab	Death	1/49	Follow-up population	Narrative	Fatal immune-mediated myocarditis during neoadjuvant treatment

Table S3. Safety definitions, perioperative outcomes, and response-assessment definitions across included studies.

(A) Safety definitions and perioperative outcomes.

Study/arm	Terminology/grading	Any-grade	Grade ≥3	Treatment interruption/discontinuation	Steroid or immunosuppression	Surgical delay/cancellation	Postoperative outcomes	Important toxicities	Pooling decision
Cercek 2025 — dostarlimab	All-cause AEs across both cohorts; not an explicit irAE dataset	65% of 124 any AE	6/124 grade 3-4	Two patients discontinued after cCR because of treatment-related AEs; other early discontinuations were not uniformly toxicity-related	NR	Curative resection remained feasible; rectal responders entered NOM	Not applicable to the nonoperative rectal pathway	Grade 3 diabetes, lung infection, hypothyroidism, encephalitis, and neutropenia; grade 4 febrile neutropenia	Exclude from pooled irAE analyses: all-cause AE framework and mixed-tumor safety population
Chalabi 2024 — nivolumab + ipilimumab	irAEs; CTCAE version NR in main report	73/115	5/115	0 discontinuations due to AEs	Immunosuppression for myositis; long-term thyroid/adrenal replacement	2/115 delays >2 weeks; no cancellation	Any grade 22/115; grade 3 12/115; anastomotic leak 4/115	Rash, enzyme elevation, myositis, hepatitis, hyponatremia	Primary analysis and strict explicit-irAE sensitivity analysis
Gooyer 2024 — nivolumab + relatlimab	Possibly drug-related AEs classified as irAEs; CTCAE v4.0; Clavien–Dindo postoperative	47/59	6/59	Second cycle omitted in 3 patients	Steroids, mycophenolate, infliximab, tacrolimus, FMT; chronic hormone replacement	3/59 delays (4–26 wk); no cancellation	Grade 3–4 postoperative AEs 4/59	Hepatitis, colitis, hyperthyroidism, nephritis	Primary analysis only; excluded from strict sensitivity because attribution was

**Study/arm**	**Terminology/grading**	**Any-grade**	**Grade ≥3**	**Treatment interruption/discontinuation**	**Steroid or immunosuppression**	**Surgical delay/cancellation**	**Postoperative outcomes**	**Important toxicities**	**Pooling decision**
									based on possibly drug-related AEs classified as irAEs
Kuznetsova 2025 — prolgolimab	Treatment-related AEs/irAEs; CTCAE v5.0	10/30	1/30	One interruption due autoimmune hepatitis	Glucocorticoid; hepatitis resolved within 1 week	Treatment-related delay/cancellation NR	Postoperative complications NR	Thyrotoxicosis, hypothyroidism, diarrhea, grade 4 hepatitis	Primary analysis only; excluded from strict sensitivity because treatment-related and immune-related attribution was mixed; postoperative safety NR
Wang 2025 — IBI310 + sintilimab	TEAE, TRAE, serious TRAE, irAE; MedDRA v26.0; CTCAE v5.0	25/52 irAE; 49/52 TEAE	2/52 irAE	1 interruption; 0 discontinuation	Not systematically summarized	2/52 delays (2 and 13 days); no cancellation	Surgery-phase TEAEs 36/52; grade ≥3 10/52; one fatal postoperative leak/sepsis	Myocarditis, enteritis	Primary analysis and strict explicit-irAE sensitivity analysis; TEAEs

Study/arm	Terminology/grading	Any-grade	Grade ≥3	Treatment interruption/discontinuation	Steroid or immunosuppression	Surgical delay/cancellation	Postoperative outcomes	Important toxicities	Pooling decision
									analyzed separately
Wang 2025 — sintilimab	TEAE, TRAE, serious TRAE, irAE; MedDRA v26.0; CTCAE v5.0	19/49 irAE; 43/49 TEAE	4/49 irAE	2 interruptions; 1 discontinuation	High-dose methylprednisolone for myocarditis	No delay; 1/49 cancellation	Surgery-phase TEAEs 34/49; grade ≥3 6/49	Fatal myocarditis, rash, hypothyroidism, ALT increase	Primary analysis and strict explicit-irAE sensitivity analysis; report treatment-related death separately

Comparability note for any-grade irAE pooling: The primary analysis included only event counts designated by the original investigators as irAEs or assigned to a study-defined immune-related attribution category. Counts reported solely as treatment-emergent, non-immune treatment-related, or all-cause adverse events were excluded. Because eligible studies still used different attribution frameworks, the post hoc sensitivity analysis was restricted to Chalabi 2024 [8] and the two Wang 2025 arms [10, 21] that explicitly reported irAEs. The pooled proportion was 0.51 (95% CI, 0.37-0.65; I^2 = 78.4%; three arms, 216 patients), consistent with the primary estimate of 0.54.

(B) Response-assessment definitions across included studies.

Study	pCR	MPR	cCR	Assessment framework	Pathology review	Implication for synthesis
Cercek 2025	Not applicable to the rectal cohort managed nonoperatively	NR	No evidence of residual disease on tumor-specific imaging and endoscopy; rectal assessment included	Tumor-specific imaging and endoscopy when feasible; biopsy used as a supportive assessment	No mandatory resection specimen; biopsy was not a substitute for the multimodal cCR assessment	cCR is a nonoperative clinical endpoint and must not be pooled with pCR; the 2025 report supersedes the 2022

Study	pCR	MPR	cCR	Assessment framework	Pathology review	Implication for synthesis
			MRI, endoscopy, and digital examination			definition/follow-up row
Chalabi 2024	0% residual viable tumor in tumor bed and lymph nodes	≤10% residual viable tumor	Not applicable	Complete resection specimen	Central review by experienced gastrointestinal pathologist	Comparable pCR/MPR definitions; central review supports consistency
Gooyer 2024	0% residual viable tumor	≤10% residual viable tumor	Not applicable	Resected primary tumor bed and lymph nodes; RVT quantified	Central histopathologic assessment	Comparable pCR/MPR definitions; central review
Kuznetsova 2025	No viable tumor cells; Mandard TRG 1	≤10% residual viable tumor; Mandard TRG 1–2	≥90% tumor shrinkage by iRECIST plus no endoscopic tumor	Radiologic/endoscopic cCR; Mandard TRG on resection specimens	Central review not clearly reported in main article	Descriptive cCR only; five of six cCR patients later underwent surgery
Wang 2025	ypT0N0 or ypTisN0 after neoadjuvant therapy	RVT ≤10%	Not an organ-preservation endpoint	All tumor and lymph-node slides after resection	Assessment at each site and the leading site	Study-specific pCR definition includes ypTisN0

Supplementary Table S4. Structured narrative certainty assessment (GRADE-informed).

Outcome	Risk of bias	Inconsistency	Indirectness	Imprecision	Publication bias	Overall certainty	Rationale
pCR	Serious	Serious	Serious	Serious	Not formally assessable	Very low	Early-phase, mostly single-arm evidence; moderate heterogeneity; five arms.
MPR	Serious	Serious	Serious	Serious	Not formally assessable	Very low	High heterogeneity; limited arms; differing assessment frameworks.
Any irAE	Serious	Very serious	Serious	Serious	Not formally assessable	Very low	Substantial heterogeneity and variable attribution; Cercek excluded.
Grade ≥3 irAE	Serious	Not serious	Serious	Serious	Not formally assessable	Very low	Few events, variable definitions, and one treatment-related death.
Surgery rate	Serious	Serious	Very serious	Serious	Not formally assessable	Very low	Protocol-driven and not a direct efficacy or organ-preservation measure.
cCR / organ preservation	Very serious	Very serious	Very serious	Very serious	Not formally assessable	Very low	Different cCR definitions, selected cohorts, and short follow-up.
Long-term outcomes	Serious	Very serious	Serious	Very serious	Not formally assessable	Very low	Immature heterogeneous DFS/EFS/OS data; no compatible hazard ratios or timepoints.

Supplementary Table S5. Follow-up and long-term oncologic outcomes.

Study/arm	Follow-up	Recurrence/progression	Survival outcomes	Deaths	Organ preservation / NOM	Durability or salvage information	Synthesis decision
Cercek 2025 — dostarlimab	Median recurrence follow-up 30.2 mo (5.8-60.8) in the rectal cohort	2-year RFS 96% (95% CI, 90-100); simple event count not separately tabulated for the rectal cohort	No deaths; median survival follow-up 21.5 mo across both cohorts	0/50	49/49 treatment completers entered NOM	37 patients had sustained cCR at 12 months; recurrence and salvage/re-treatment were reported across the combined study cohorts	Narrative; mature 2025 report replaces the 2022 cCR, NOM, and follow-up data and is not counted as an additional study
Chalabi 2024 — nivolumab + ipilimumab	26 mo (9–65)	0/115	3-year DFS primary endpoint not mature	NR	All underwent protocol-mandated surgery	Not applicable	Narrative recurrence summary
Gooyer 2024 — nivolumab + relatlimab	8 mo (2–19)	1/59; 58/59 disease-free	DFS/OS immature; all alive	0/59	All underwent surgery	Not applicable	Narrative because follow-up is short
Kuznetsova 2025 — prolgolimab	19 mo (13–32)	2 confirmed progressions	18-month DFS and OS both 90%	3/30	Three refused surgery: one cCR and two partial clinical responses	Tumor control reported; exact patient-level NOM duration and salvage outcomes not fully tabulated	Narrative; progression/death definitions differ from other studies
Wang 2025 — IBI310 + sintilimab	21.4 mo (1.5–24.6)	0/52	One EFS/OS event; outcomes immature	1/52	Not evaluated as organ preservation	Postoperative death after anastomotic leak and septic shock	Narrative; adjuvant treatment was not standardized

Study/arm	Follow-up	Recurrence/progression	Survival outcomes	Deaths	Organ preservation / NOM	Durability or salvage information	Synthesis decision
Wang 2025 — sintilimab	21.4 mo (1.5–24.6)	0/49	One EFS/OS event; outcomes immature	1/49	Not evaluated; surgery canceled in fatal myocarditis case	No salvage surgery reported	Narrative

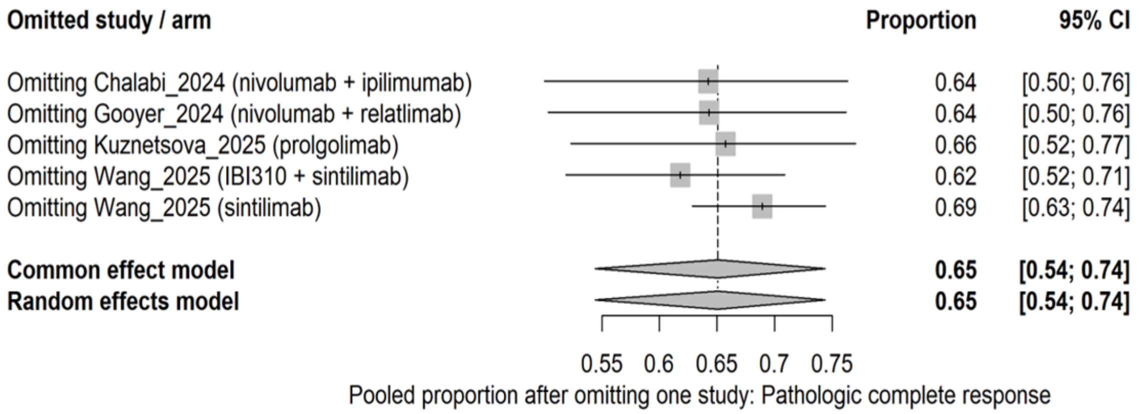

Figure S1. Leave-one-out sensitivity analysis for pathologic complete response (pCR).

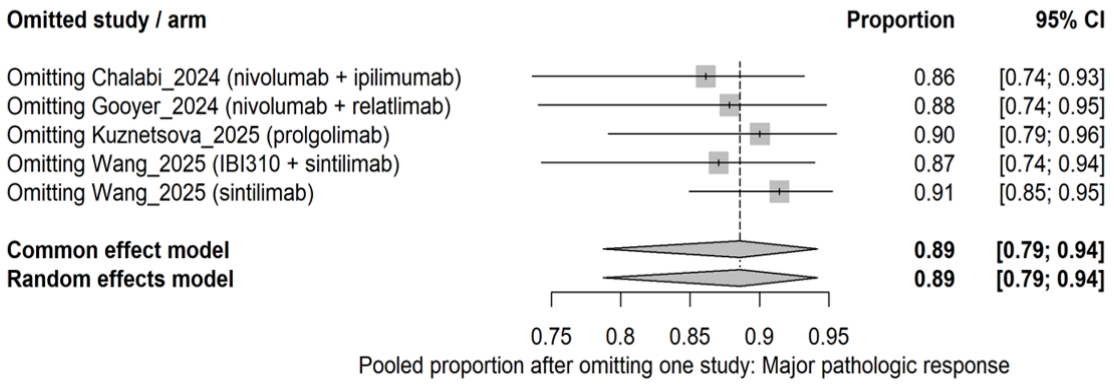

Figure S2. Leave-one-out sensitivity analysis for major pathologic response (MPR).

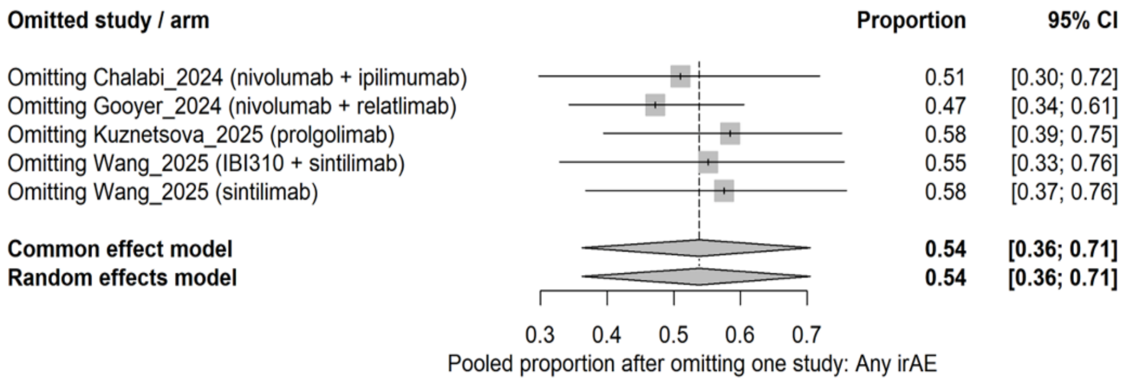

Figure S3. Leave-one-out sensitivity analysis for any immune-related adverse event (irAE).

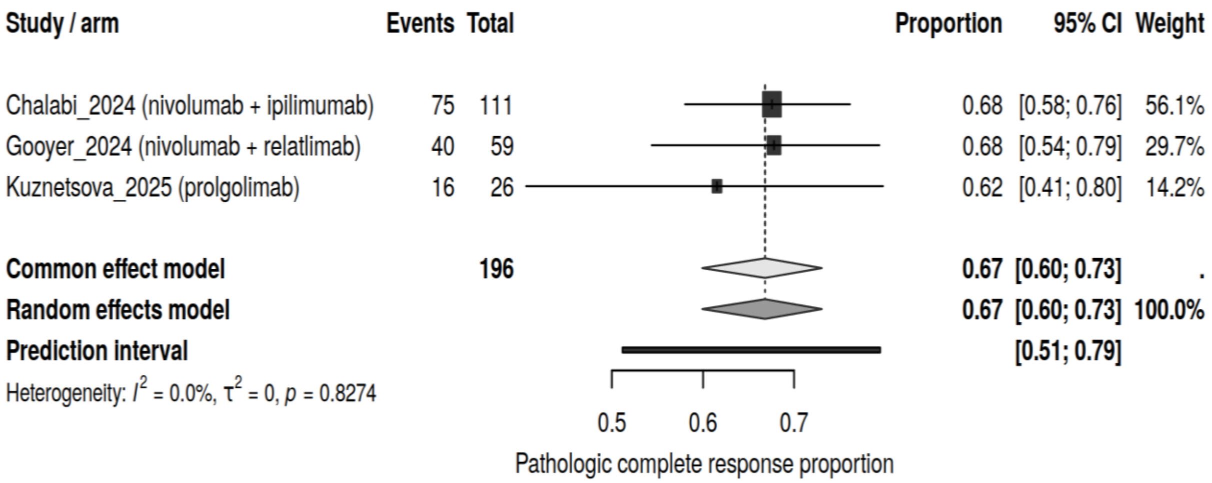

Figure S4. Sensitivity analysis excluding the randomized phase 1b Wang 2025 trial [10], which was also the study judged at high risk of bias, for pathologic complete response (pCR).

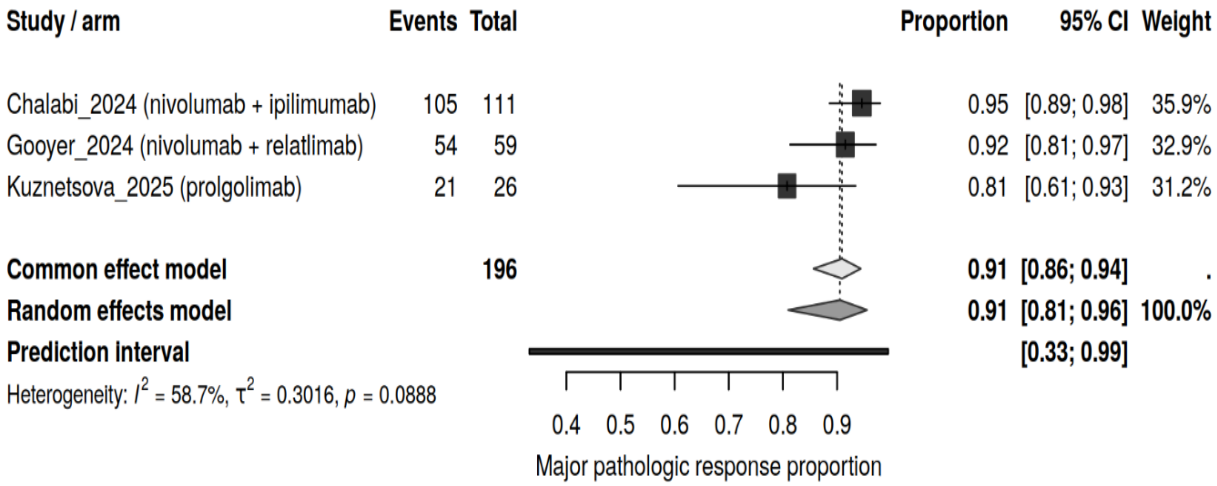

Figure S5. Sensitivity analysis excluding the randomized phase 1b Wang 2025 trial [10], which was also the study judged at high risk of bias, for major pathologic response (MPR).

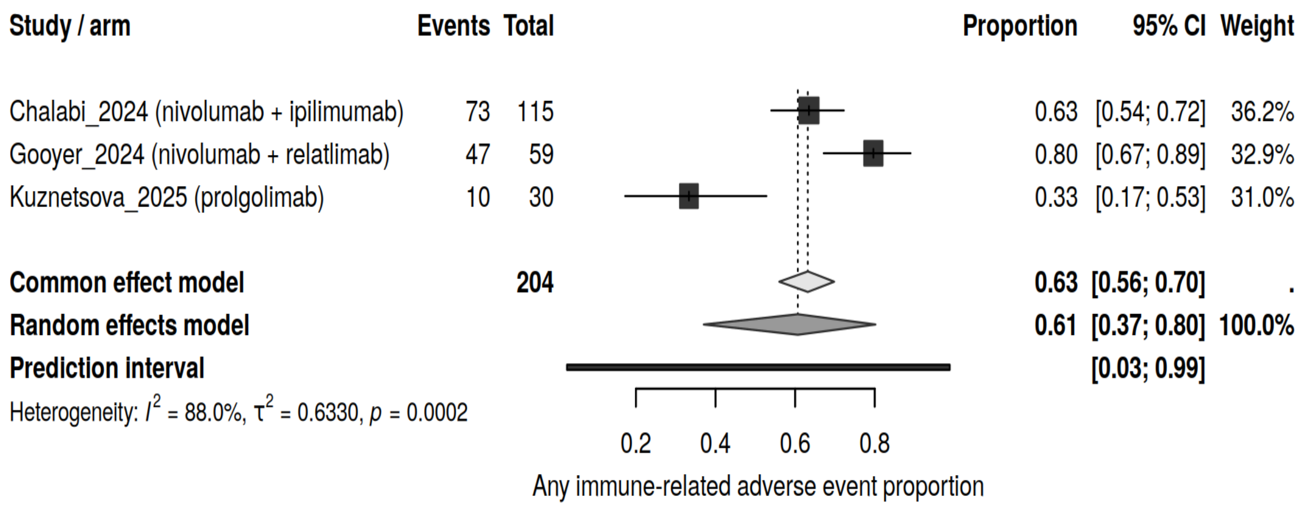

Figure S6. Sensitivity analysis excluding the randomized phase 1b Wang 2025 trial [10], which was also the study judged at high risk of bias, for any immune-related adverse event (irAE).

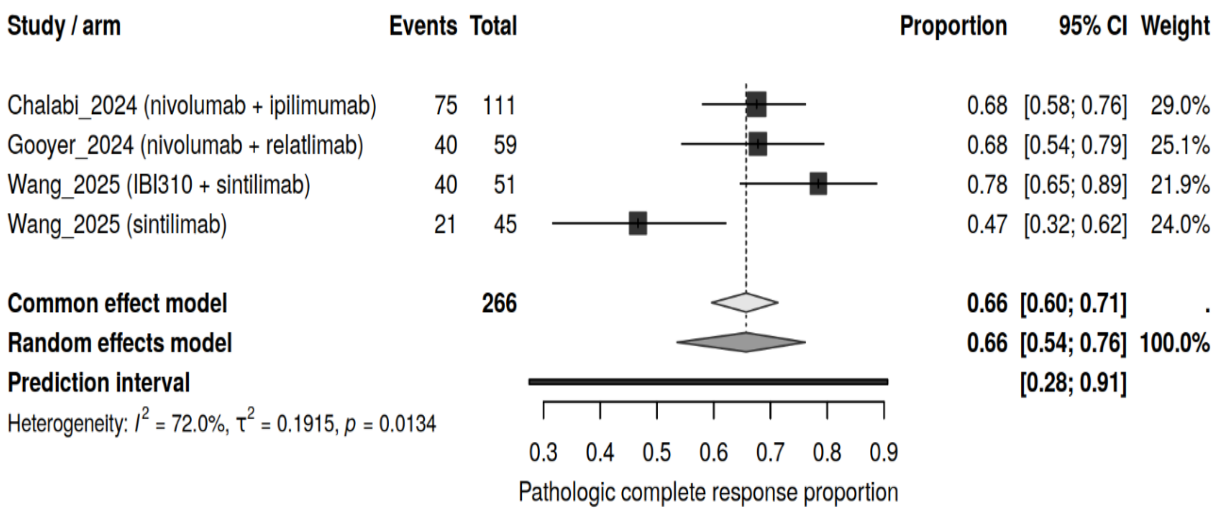

Figure S7. Colon-only sensitivity analysis excluding the mixed colon/rectal Kuznetsova 2025 cohort [11] for pathologic complete response (pCR)

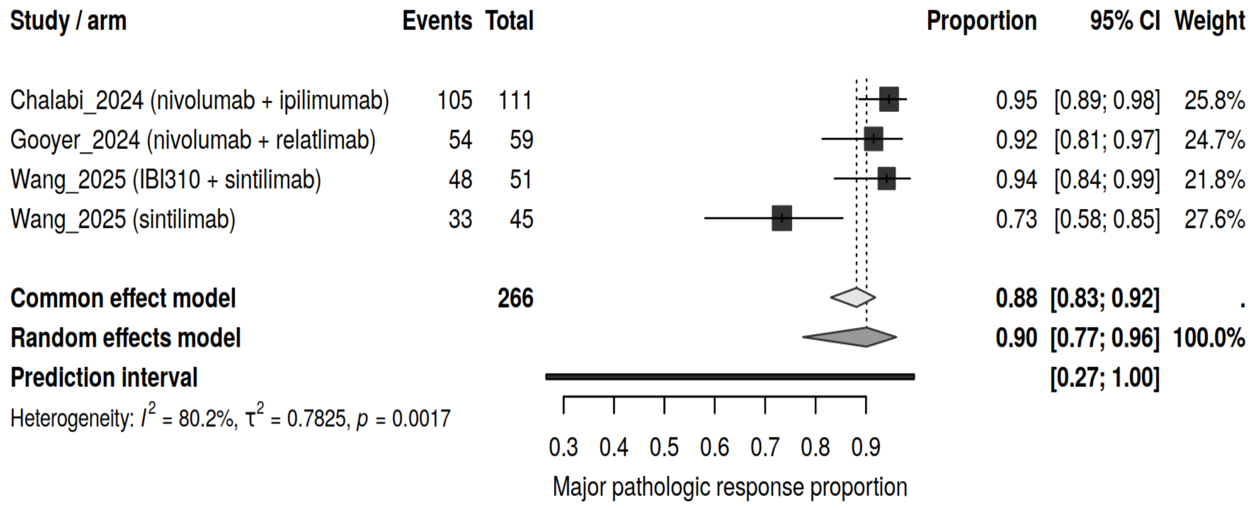

Figure S8. Colon-only sensitivity analysis excluding the mixed colon/rectal Kuznetsova 2025 cohort [11] for major pathologic response (MPR).

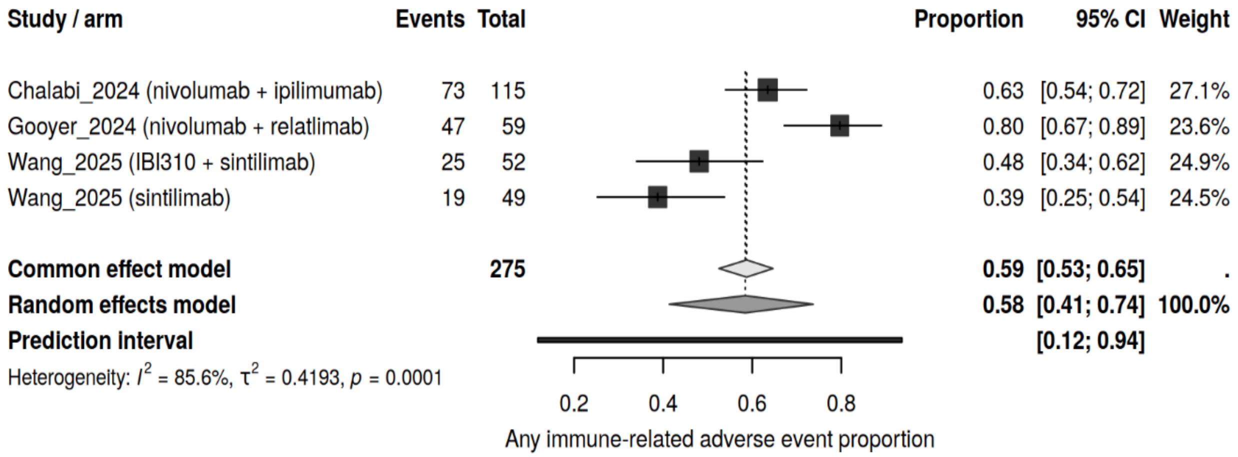

Figure S9. Colon-only sensitivity analysis excluding the mixed colon/rectal Kuznetsova 2025 cohort [11] for any immune-related adverse event (irAE)
